# Characterization, Performance, and Toxicological Assessment of Polysulfone-Sulfonated Polyether Ether Ketone Membranes for Water Separation Applications

**DOI:** 10.3390/membranes15030087

**Published:** 2025-03-08

**Authors:** Muhammad Usman Yousaf, Lucca Madeo Cortarelli, Nerissa I. Jebet, Jason M. Unrine, Nirupam Aich, Olga V. Tsyusko, Isabel C. Escobar

**Affiliations:** 1 Department of Chemical and Materials Engineering, Stanley and Karen Pigman College of Engineering, University of Kentucky, Lexington, KY 40506, USA; usman.yousaf@uky.edu; 2 Department of Plant and Soil Sciences, Martin-Gatton College of Agriculture, Food and Environment, University of Kentucky, Lexington, KY 40546, USA; lucca.cortarelli@uky.edu (L.M.C.); jason.unrine@uky.edu (J.M.U.); 3 Department of Health and Clinical Sciences, College of Health Sciences, University of Kentucky, Lexington, KY 40506, USA; njki227@uky.edu; 4 Kentucky Water Research Institute, University of Kentucky, Lexington, KY 40506, USA; 5 Department of Civil and Environmental Engineering, College of Engineering, University of Nebraska–Lincoln, Lincoln, NE 68588, USA; nirupam.aich@unl.edu

**Keywords:** polymeric membranes, membrane toxicity, annealing, non-solvent induced phase separation, functionalization, safe(r)-by-design, permeability, solvent leaching, water separation/filtration

## Abstract

The removal of small molecular weight charged compounds from aqueous solutions using membrane remains a challenge. In this study, polysulfone (PSf)- and sulfonated polyether ether ketone (SPEEK)-based membranes were fabricated via non-solvent induced phase separation process (NIPS) using N-Methyl-2-Pyrrolidone (NMP) as solvent and water as non-solvent. Membranes were characterized structurally and morphologically, followed by toxicity assessment conducted before and after filtration, both with and without annealing at various pH values to evaluate potential leaching of trapped solvent from the membrane pores. Additionally, membrane performance was characterized using binary mixtures of cationic and anionic dyes. The results demonstrated selective filtration behavior, with cationic dyes being preferentially rejected due to size exclusion and electrostatic interactions. Additionally, a key focus of this work was the investigation of solvent leaching, framed within a Safe(r)-by-Design (SbD) approach aimed at enhancing functional performance while minimizing environmental toxicity. Toxicity assessments using a model organism, a nematode *Caenorhabditis elegans*, revealed that annealing reduced solvent leaching and thus permeate toxicity, particularly at neutral pH values, by facilitating trapped solvent release prior to membrane use. These findings provide insights for the importance of including an SbD approach during membrane casting to fabricate membranes with desirable properties while minimizing toxicity.

## 1. Introduction

High-efficiency separation processes are increasingly desired for production of high-purity, high-value products in applications associated with water treatment, the pharmaceutical and food sectors [1]. Compared to traditional separation technologies, membranes offer distinct advantages, including lower manufacturing cost, reduced energy consumption, and operational flexibility. Membrane fabrication necessitates high permeability and selectivity while minimizing effluent toxicity to ensure a Safe(r)-by-Design (SbD) approach in applications involving water treatment [2]. The current research focus includes development of novel materials and processes to tackle challenging separations, such as the removal of emerging contaminants, per- and polyfluoroalkyl substances (PFAS), through multifunctional membrane processes [3]. However, a significant gap remains regarding toxicity investigations associated with membrane fabrication, especially those employing emerging high-performance polymers for difficult separations.

Membrane fabrication often utilizes non-solvent induced phase separation (NIPS) technique in which dope solution, made of a polymer dissolved in a solvent, is cast on a solid surface and immersed in a non-solvent coagulation bath, mainly water [4]. Kinetic and thermodynamic factors are involved in membrane structure formation which determine interfacial stability, and interactions among the polymer, solvent, and non-solvent. These parameters are influenced by material selection, the dope solution polymer composition, polymer chemistry, and post-treatment processes which significantly affect the membrane’s final structure. These variations influence the membrane’s morphology and its separation performance [5]. For example, the incorporation of functional groups in polymer matrix modifies surface chemistry, which influences membrane properties, such as hydrophilicity [6], and casting dope solution concentrations control pore size and distribution during membrane formation due to changes in thermodynamic and kinetic properties of the system [7]. Therefore, polymer chemistry and optimization of dope solution concentration remains crucial for membrane tuning towards specific applications.

Polysulfone (PSf) is a well-studied polymer in ultrafiltration and nanofiltration membrane fabrication due to its mechanical properties and physicochemical stability over a wide pH range [8]. Despite these advantages, the nonpolar benzene rings of PSf increase hydrophobicity which reduce water flux and increase membrane propensity to fouling [8]. To overcome the challenge of hydrophobicity, membranes have been surface-functionalized through CO_2_ plasma treatment [9], UV surface grafting [10,11], and surface coating [12]. However, these techniques encompass cumbersome procedures and scalability. Polymer blending with charged polymers offers a promising alternative, to enhance hydrophilicity and impart charge to the membrane [13]. In this study, the high-performance polymer polyether ether ketone (PEEK) is proposed for functionalization with sulfonic groups and subsequent blending with PSf to increase hydrophilicity and charge. PEEK is a high-performance thermoplastic with thermal stability, and chemical resistance [14]. The incorporation of sulfonic acid groups (-SO_3_H) to develop sulfonated polyether ether ketone (SPEEK) introduces charged groups to its structure, which makes it hydrophilic, enhances the ion-exchange capacity, and makes it soluble in polar aprotic solvents. The degree of sulfonation (DS) can be controlled by time and temperature of the reaction which allows to customize polymer for water treatment applications [15].

Polymeric membranes may retain solvents used in the casting process, such as N-methyl-2-pyrrolidone (NMP). Despite this drawback, the reasons NMP was utilized in this study for membrane casting include its strong dissolving power, low vapor pressure, and high relative boiling point of 202 °C [16]. Moreover, NMP was selected due to its ability to dissolve both PSf and SPEEK which provided a balanced combination of polymer solubility, and controlled phase separation in the final membrane structure. Although widely utilized, NMP requires special safety measures due to the European Union’s (EU) Registration, Evaluation, Authorization, and Restriction of Chemicals (REACH) regulation [17].

Membranes may leach solvent trapped within membrane pores during filtration, leading to solvent release into effluents. To address this, post-fabrication, polymeric membranes undergo a thermal annealing process, which allows any trapped solvent to be released due to changes in membrane structure [18]. Annealing improves film morphology and enhances interchain interaction of polymeric chains leading to more compact membranes, especially in polymer blends, conjugated polymers, and ion-conducting polymers [19]. Annealing effects on SPEEK membranes were studied at temperatures between 120 and 160 °C. This promoted cross-linking through SO_2_ bridges between polymer chains which significantly reduced water uptake, and minimized swelling [20]. Before annealing, membranes may have trapped solvent in their pores. It is likely that the NMP solvent will be released due to reduction in the membrane pore size. Thus, annealing may prove beneficial for tailored membrane performance in specific applications while simultaneously reducing toxicity of solutions filtered through them. Consequently, annealing may provide a promising Safe(r)-by-Design (SbD) strategy to optimize membrane performance for specific applications while reducing the toxicity of filtered permeates.

Potential origins of toxicity in membrane fabrication and usage require identification particularly relating to solvents such as NMP, which can leach from membranes into permeates. SbD approaches are crucial for toxicity assessment at all stages of polymeric membrane development. Toxicity assessments rely on a model nematode, *Caenorhabditis elegans*, chosen for its high reproductive capacity, short generation time, and fully sequenced and annotated genome [21]. *C. elegans* are essential to meet the National Institutes of Health’s (NIH) goals for refining, reducing, and replacing vertebrate animal testing in toxicology. Toxicity ranking screens have shown high predictive potential for rats and mice, and various modes of toxic action conservation have been identified between mammals and *C. elegans* [22]. Additionally, *C. elegans* shares 60–80% of protein-coding genes that are homologous to humans [23] underscoring its significance as a model organism for toxicity testing in SbD product development.

This study explores the evolution of polymer blends, specifically the blending of PSf and SPEEK for the fabrication of NF membranes. By systematically varying the composition of the polymer blend, the study aimed to optimize the membrane’s hydrophilicity, mechanical strength, and filtration efficiency while simultaneously reducing toxicity. Thus, the focus is on controlling membrane properties while minimizing toxicity through a SbD approach, the area which is currently unexplored. Filtration experiments were conducted to evaluate the preliminary performance of membranes using various dyes with different molecular sizes and charges. Toxicity assessments were conducted following the filtration of solutions at varying pH to guide membrane operation, maximizing performance while minimizing environmental impacts. Overall, the findings contribute to advancing the understanding of membrane design for efficient and environmentally benign separation processes.

## 2. Materials and Methods

### 2.1. Synthesis of Sulfonated PEEK (SPEEK) and Membrane Fabrication

**Figure 1:**
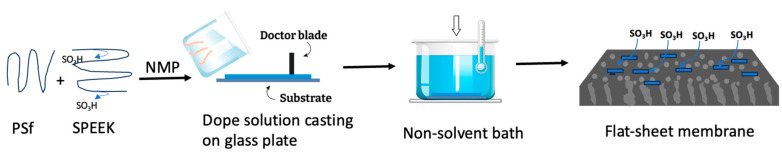
Flat Sheet Membrane synthesis via Non-Solvent Induced Phase Separation (NIPS).

All reagents used in the synthesis and characterizations are of analytical grade and do not require additional purification unless stated otherwise. All materials used are listed in Supplementary Materials S.2. PSf and SPEEK were used to fabricate membranes using NIPS method. SPEEK was synthesized by sulfonating polyether ether ketone (PEEK) in concentrated sulfuric acid, as described in detail in S.3.1, with the degree of sulfonation (DS) optimized for membrane fabrication. In this study, the DS was controlled at 50–60% to ensure a balance between hydrophilicity and chemical stability. Membrane dope solutions were prepared as described in S.3.2, with varying overall polymer concentrations by blending 5 wt.% SPEEK in N-methyl-2-pyrrolidone (NMP) and stirring until complete dissolution. Once SPEEK was fully dissolved, polysulfone (PSf) was then added to the solution under continuous stirring to ensure homogeneous dope solution. The solutions were degassed, cast onto glass plates, and phase-inverted using deionized water to form asymmetric membranes. The pictorial representation of the fabrication procedure is illustrated in Figure 1 and discussed in S.3.3. To fabricate the membranes under SbD approach, post-treatment annealing was performed using two different approaches: oven annealing and water annealing. Under various testing conditions (e.g., pH), NMP should not leach from fabricated membranes [24]. Thus, assessing membrane toxicity at various pH is essential to ensure limited NMP leaching. Addition of the thermal annealing step is our proposed method to minimize NMP release from membranes according to a Safe(r)-By-Design approach to membrane development. For water annealing, membranes were immersed in deionized water at 80 °C for 45 min. For oven annealing, membrane (PSN-19) was treated in pre-heated oven at 120 °C for 1 min. The annealing temperature was maintained below the glass transition temperature of the polymers to prevent structural degradation. Membranes with 17% and 19% PSf were labeled as PN-17 and PN-19, respectively. Following SPEEK addition, membranes with 17,19, 21, and 23 wt.% dope solutions were designated as PSN-17, PSN-19, PSN-21, and PSN-23. The oven-annealed and water-annealed membranes were labeled as PSN-19O and PSN-19W, respectively.

### 2.2. Membrane Characterization

Details on membrane characterization techniques are provided in the Supplementary Materials S.4. The DS of SPEEK was determined via hydrogen nuclear magnetic resonance (H-NMR) spectroscopy (S.4.1). Cloud points were determined to assess the thermodynamic stability of polymer solutions, while viscosity was measured using a digital rheometer, as described in S.4.3 and S.4.4. Chemical functionality of SPEEK and membrane surface was analyzed using FTIR (S.4.5), while SEM (S.4.6) was employed for surface and cross-sectional morphology. XPS (S.4.7) provided insights into elemental composition. Hydrophilicity was assessed through sessile drop water contact angle measurements (S.4.8), and porosity was determined using a gas pycnometer (S.4.9). Mechanical strength was tested using a tensile stress analyzer (S.4.10) to measure ultimate tensile strength.

### 2.3. Toxicity Assays

These were conducted in MHRW. Moderately Hard Reconstituted Water (MHRW), a standard EPA toxicity medium, was prepared using the following concentrations: 0.00114 M NaHCO_3_, 0.000348 M CaSO_4_·2H_2_O, 0.000497 M MgSO_4_·7H_2_O, and 0.000054 M KCl [25,26]. pH was then adjusted through titration with sulfuric acid or sodium hydroxide. Wild-type N2 nematodes were utilized. An unfiltered control was utilized, and treatments involved solutions from recycling filtration five times through PSN-19 and PSN-19W membranes. All experiments relied on age-synchronization of nematodes and acclimation to low ionic strength MHRW. Mortality experiments involved 24-h exposures in 24-well plates with no bacterial food supply. A reference toxicant (CdCl_2_ at LC_50_ of 21 mg/L) was included in each mortality assay. Reproduction experiments involved 48-h exposures in MHRW followed by 72-h reproduction in K-agar plates with *E. coli* OP50 bacterial food supply. The experimental design for mortality and reproduction followed previously established protocols described in Supplementary Information S4.11, S4.12, S4.14, and S4.14. Filtration of solutions are described in Supplementary Information S4.13 and statistical analysis for results is described in Supplementary Information S4.16.

### 2.4. Membrane Performance

Details regarding membrane operation performance are summarized in Supplementary Information S.4.17. Filtration experiments were conducted at a constant pressure of 4.14 bar (60 psi). Prior to testing, membrane samples (effective area: 14.6 cm^2^) were pre-compacted with DI water at a pressure of 4.14 bar (60 psi) to stabilize flux and minimize compaction effects. The experiments were performed using aqueous solutions of organic dyes with different molecular weights, charges, and radii including Congo Red (CR), Methylene Blue (MB), Crystal Violet (CV), and Acid Orange 2 (AO2). Binary dye mixture (MB:AO2, 50:50) was also tested. The properties of the dyes are given in Table S1.

## 3. Results and Discussion

### 3.1. Characterizations of Synthesized SPEEK

**Figure 2:**
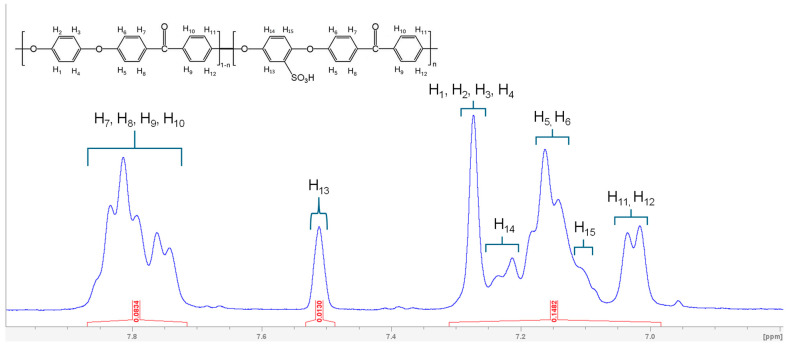
H-NMR spectrum of SPEEK alongside the nomenclature of SPEEK repeating units.

The structure and H-NMR spectrum of SPEEK, shown in Figure 2, highlight the chemical shifts corresponding to aromatic protons. It is important to measure the DS because SPEEK with high DS has relatively low chemical stability [27]. Moreover, DS affects SPEEK solubility in solvents [15]. Therefore, it is essential to control and measure the DS of synthesized SPEEK to utilize it further for membrane fabrication. The sulfonation of PEEK introduces electron-withdrawing sulfonic acid groups (-SO_3_H) onto its aromatic rings, transforming it into SPEEK. This modification disrupted the electron density surrounding the originally equivalent protons (H_1,2,3,4_) that appear as a single peak at 7.26 ppm to differentiate to three categories H_13_ (at 7.5 ppm), H_14_ (at 7.22 ppm), and H_15_ (at 7.11 ppm), due to electron withdrawing effect of sulfonic acid groups. The peaks were confirmed from the literature, and DS calculated using eq. 1 [28,29]. This ratio between peak area of distinct H_13_ signal and the integrated area of all other peaks from H_1_ to H_15_ excluding H_13_ gave a DS of 57%. The introduction of sulfonic acid groups made polymer soluble in aprotic solvents too, which allowed the fabrication of membranes membrane via NIPS.

(1) $$\frac{DS}{12-2DS}=\frac{S_{H13}}{S_{H(1,2,3,4,5,6,7,8,9,10,11,12,14,15)}}$$

**Figure 3:**
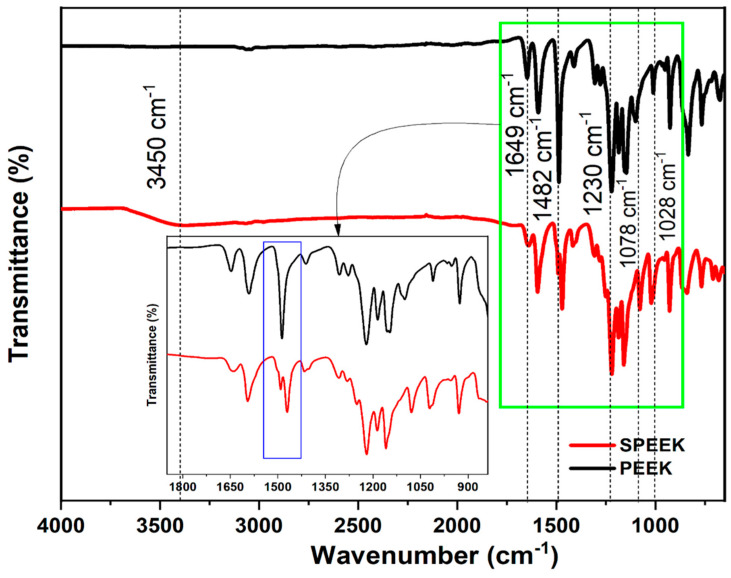
FTIR spectra showing the functional peaks comparison of PEEK and synthesized SPEEK (sub-figure (green selection) highlights the formation of shoulder band in SPEEK).

FTIR spectra were used to confirm changes in surface functional groups of PEEK and SPEEK polymer. Figure 3 shows the spectra of PEEK and SPEEK, where the absorption peaks at 1028 cm^−1^ and 1078 cm^−1^ correspond to the symmetric O=S=O and asymmetric stretch of S=O from SO_4_^3-^ groups, respectively, which were attached from the sulfonation reaction. The absorption at 1649 cm^−1^ was assigned to the carbonyl group (C=O) which appeared in the spectrum of SPEEK. A change in absorption peak of aromatic C-C at 1482 cm^−1^ of PEEK was observed after the introduction of sulfonic acid group in PEEK backbone. The single characteristic peak at 1482 cm^−1^ in PEEK changed to a shoulder band in SPEEK which shows the substitution of sulfonic acid group in the aromatic C-C ring [28]. Additionally, the presence of C-O-C structure was identified at 1230 cm^−1^. These peaks agree with the literature [30].

### 3.2. Dope Solution Behavior: Cloud Point and Viscosity

Cloud points were determined to obtain a ternary phase diagram of polymer/solvent/nonsolvent systems, as discussed in Supplementary Information S5.1 and shown in Figure S2. This was used to explore the thermodynamic behavior of the dope solution to determine the compositional path for membrane precipitation. The precipitation points of PSf and PSf-SPEEK solutions using water as a non-solvent is shown in Figure S2. SPEEK addition to the PSf matrix (PSf-SPEEK) in the casting solutions shifts the cloud point curve (shown in green), indicating that less water is required to induce precipitation. This suggests that solutions become less thermodynamically stable following SPEEK addition. The cloud point study showed that SPEEK addition reduced the amount of water required for phase separation, suggesting increased affinity between the polymer and nonsolvent, which promotes earlier phase separation. This behavior is attributed to the higher water-uptake of SPEEK, which enhances water–polymer interactions, leading to faster demixing during the phase inversion process.

Dope solution viscosity impacts dissolution kinetics and membrane structure during phase inversion. As shown in Figure S3, viscosity increased with polymer concentration due to chain entanglement. SPEEK-based solutions exhibited higher viscosities than PSf membranes, indicating additional molecular interactions.

### 3.3. Characterization of Fabricated Membranes

FTIR spectra, shown in Figure S4, confirmed the surface functional groups of fabricated membranes. PSf and SPEEK share some functional groups, but additional peaks at 1649 cm^−1^ and 1052 cm^−1^ were observed in the PSf-SPEEK membrane, corresponding to the C=O and S=O from SO_4_^3-^ groups, respectively, confirming the incorporation of SPEEK into the PSf matrix. This is observed in the literature incorporating SPEEK with another polymer [31,32]. The introduction of SPEEK was further confirmed by XPS. XPS analysis of pristine PSf (PN-19), and PSf-SPEEK (PSN-19) membranes, shown in Table S2 and Figure S5, revealed changes in elemental composition and chemical environments with blending. The decrease in carbon content and increase in oxygen in PSN-19 confirm the introduction of sulfonated SPEEK. Annealing further induced shifts in binding energies and changes in elemental percentages, possibly due to cross-linking, or reorganization of the sulfonated domains, which may have enhanced the interaction between PSf and SPEEK.

**Figure 4:**
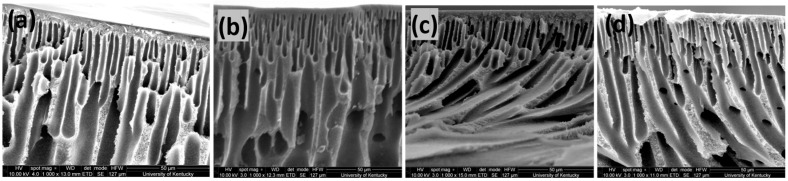
SEM cross-sectional images at 1000× (**a**) PN-19, (**b**) PSN-19, (**c**) PSN-21, (**d**) PSN-23. Cryogenic fractured using liquid nitrogen.

Cross-sectional morphologies of PN-19, PSN-19, PSN-21 and PSN-23 are shown in Figure 4. The formation of asymmetric membranes is observed in all the images which favors water permeation due to improved selectivity from a dense surface and minimum resistance from porous layer [33]. At higher dope concentrations, membrane skin layers were denser than those in membranes with lower dope solution concentrations. In Figure 4b, PSN-19 depicts a thinner skin layer compared to PSN-21 in Figure 4c. In Figure 4d, PSN-23’s skin layer was spongier compared to PSN-19 and PSN-21. Morphologies of both surfaces and cross-sections of PSN-17 and PSN-23 are shown in Figure S6. PSN-17 membranes depict a porous surface. SPEEK addition enhanced the membrane porosity by accelerating the phase inversion process due to SPEEK’s hydrophilic nature, which enhanced the solvent-nonsolvent diffusion. Consequently, larger pores are formed resulting in a more permeable structure. A similar rational has been employed in literature to explain the mechanism by which casting solution viscosity affects the process [34,35,36]. In contrast, in PSN-23, shown in Figure S6b, the surface became denser with fewer visible pores, and no pores were visible at lower magnifications. This dense structure is due to the increased viscosity of the casting solution at higher dope solution concentrations, which slowed down the demixing process, during the phase inversion process [37]. Overall, SPEEK enhanced the pore size, but higher dope solution viscosity balanced the membrane structure. The dope solution concentration did not affect the sub-surface structures as all membranes showed finger-like structures during phase inversion. It is evident from previous studies that macrovoids and finger-like structures are observed when the demixing process is rapid [32,34].

The void volume fraction of the membranes was determined by measuring total porosity using a gas pycnometer, which are listed in Table 1. Pristine polysulfone membranes (PN-17 and PN-19) showed relatively low porosity, but SPEEK incorporation significantly increased the porosity. Modified membranes exhibited an average total porosity of >75%. The addition of hydrophilic sulfonic acid functional groups and oxygen-rich functionality imparted from SPEEK increased membrane porosity due to increased dope solution polarity. The cloud point study revealed that SPEEK addition reduced the amount of water required for phase separation, indicating that the system is thermodynamically less stable. This accelerated the diffusion process of solvent outflow and non-solvent inflow, leading to increased porosity. A similar observation was obtained by Dalmini et. al., where dicarboxylic acid addition in dope solutions hastened the separation process resulting in increased porosity [38]. Wang et al. used SPEEK as a polyelectrolyte to prepare multilayer films and addition of these ionic functional group enhanced the diffusion processes of solvent outflow and non-solvent inflow to create more porous voids [38,39].

The tensile strength of membranes at different polymer concentrations before and after incorporation of SPEEK are reported in Table 1. An increase in polymer concentration led to an increase in membrane tensile strength, due to greater polymer chain density. However, SPEEK incorporation into the PSf matrix reduced the mechanical strength, as observed by PN-17 and PN-19 versus PSN-17 and PSN-19. This reduction is due to increased porosity caused by thermodynamic instability after SPEEK incorporation which was by porosity measurements. Despite this, higher polymer concentrations reduced mechanical strength loss, as seen in PSN-21 and PSN-23 membranes, where the increased polymer chain density compensated for structural compromises introduced by higher porosity. Porosity of water-annealed membranes (PSN-19W, 79.4%) and oven-annealed membranes (PSN-19O, 78.9%) was lower than that of PSN-19 (82.1%) which influenced mechanical strength as well. This porosity decrease can be attributed to the annealing process, which may have altered polymer chain packing and resulted in a slightly more compact structure. However, the changes were not significant enough to cause a major deviation in membrane performance.

**Figure 5:**
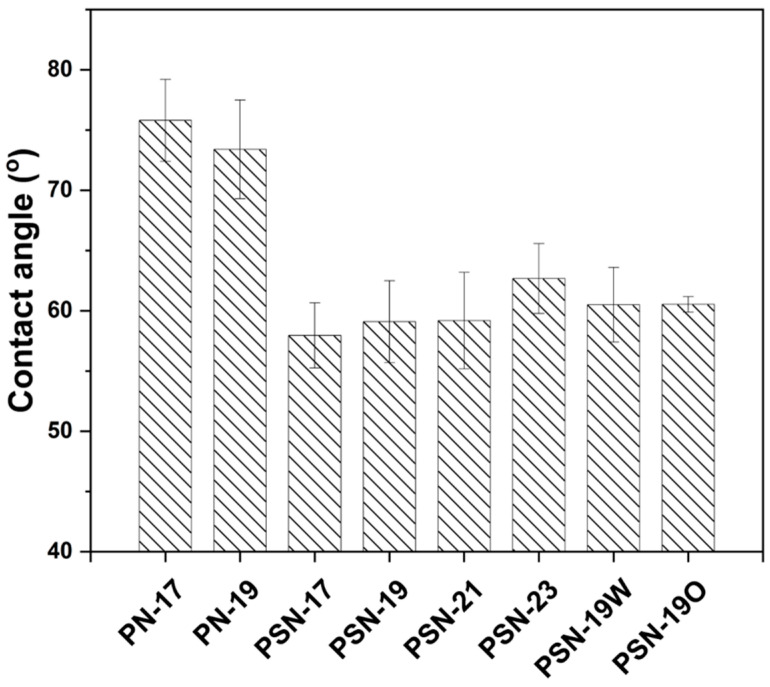
Contact angle measurements of fabricated membranes (PN-17, PN-19, PSN-17, PSN-19, PSN-23, PSN-19W, PSN-19O) to analyze hydrophilicity.

The contact angle measurements reflect membrane hydrophilicity, determined by intermolecular interactions between the membrane surface and water droplets. This is strongly related to water permeance through the membranes [40]. Measured contact angle values are shown in Figure 5. The contact angle of pristine PSf membranes were the highest for PN-17 (75.8° ± 4.1) and PN-19 (73.4° ± 3.4) dope solutions, respectively, which showed the relatively hydrophobic behavior of membranes compared to SPEEK blended membranes. The hydrophobicity of PSf is attributed to its amorphous structure from non-polar benzene rings [41]. The contact angle of membranes was reduced with SPEEK addition to 57.9° in PSN-17, 59.1° in PSN-19 and 62.67° in PSN-23. Membranes blended with SPEEK contain a ketone functional group. The oxygen-rich functionality reduced the membrane’s contact angle and made it slightly less hydrophobic [31]. Moreover, SPEEK addition imparted an ionic character to the membranes through polar sulfonic acid functional group addition which enhanced the presence of the water-channel pathways inside the membrane structure and helped increase hydrophilicity. Moreover, the contact angle decrease indicated successful incorporation of SPEEK inside the PSf matrix. This reduction of contact angle after addition of SPEEK in polymer matrix has been observed previously and it is also influenced by the DS of PEEK [32]. Additionally, the exact reduction depends not only on DS but also on fabrication conditions, such as solvent choice, phase inversion kinetics, and thermal treatment, which can further impact the final contact angle values. All SPEEK blended membranes showed comparable contact angles, because the materials were the same, and slight changes are attributed to decreased porosity with increasing dope solution concentration. A change was observed after thermal annealing of membranes, 60.5° and 60.6° in PSN-19W and PSN-19O, respectively. The observed difference in contact angles after thermal annealing is slight and it is within the measurement error range. The change in contact angle after annealing has been reported in previous studies, where changes in contact angle after thermal treatment were attributed to increased packing density of polymer chains due to improved polymeric chain arrangements or solvent removal, which hinders water penetration into the membrane surface [42].

### 3.4. Toxicity Assessments

#### 3.4.1. NMP Toxicity (Mortality and Reproduction)

PSN-19 and PSN19W were selected for these assays based on membrane characterization data (porosity, strength, and hydrophilicity) and membrane performance as described in Section 4.

**Figure 6:**
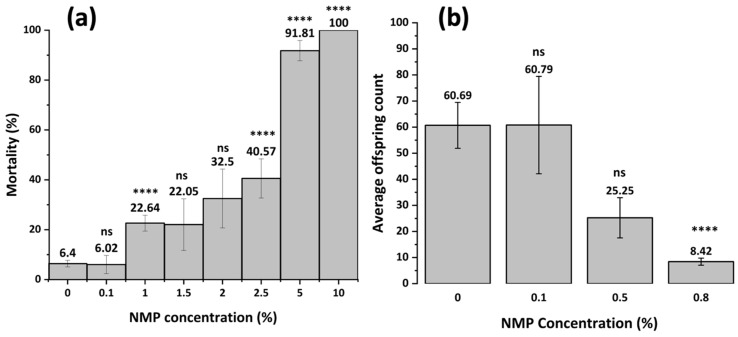
Effect of NMP on *C. elegans* mortality (**a**) and reproduction (**b**) at varying pH for 24 h without feeding and 72 h with feeding, respectively. Non-statistically significant comparisons to controls are indicated with ns at *p* > 0.05. The asterisks indicate significance at *p* < 0.0001.

Figure 6 illustrates concentration-response relationship between mortality (a) or reproduction (b) with NMP with the median lethal NMP concentration, LC_50_, at 2.39% and the median effective concentration, EC_50_ for reproduction, at 0.60% and EC_10_ at 0.04%. This indicates that NMP in solution post-filtration should not exceed 0.04% to minimize toxicity.

#### 3.4.2. Mortality Following Filtration of MHRW at Varying pH

**Figure 7:**
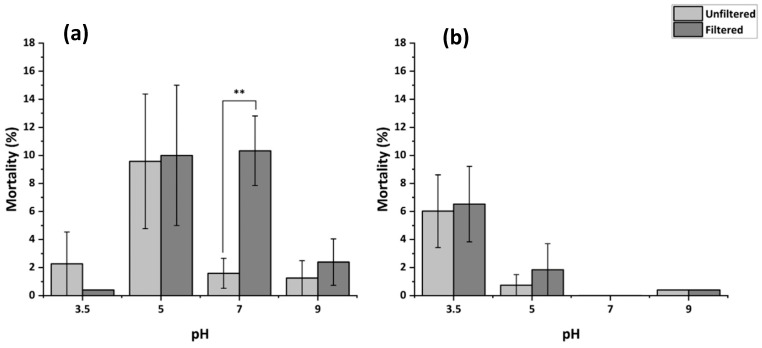
Toxicity of filtrates at varying pH through unannealed PSN-19 (**a**) and annealed PSN-19W (**b**) towards *C. elegans* exposed for 24 h without feeding. The asterisks indicate statistical significance at *p* < 0.01 after filtration compared to their respective unfiltered control.

As shown in Figure 7a, 5× filtration of pH 7 permeates through PSN-19 results in significant increase in mortality. This increase in toxicity is attributed to potential fouling, whereby NMP solvents used in the membrane fabrication are released into the solution. Meanwhile, Figure 7b illustrates the results from nematodes exposed for 24 h to pH-adjusted MHRW before and after 5× filtration through annealed PSN-19W. Toxicity increase was not observed after filtration at pH 7 for PSN-19W indicating that annealing may decrease pore size while minimizing potential NMP solvent leaching. There was no significant difference in mortality between the unfiltered controls at pH 3.5 which are shown in Figure 7a,b. Because two different set of membranes (unannealed and annealed) were used when testing membrane toxicity after filtration, toxicity comparisons of the filtered MHRW were performed with their respective controls at each tested pH (Figure 7a,b). There was no significant change in mortality observed at pH 3.5, pH 5, and pH 9 after filtration for both unannealed and annealed membranes.

#### 3.4.3. Reproduction Following Filtration of MHRW at Varying pH

**Figure 8:**
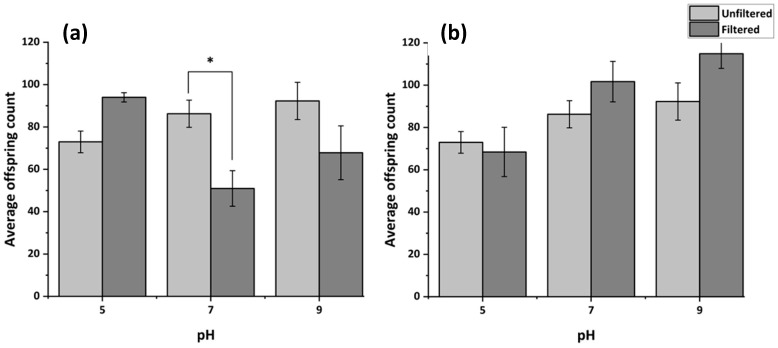
Reproductive toxicity of MHRW at varying pH filtered through PSN-19 (**a**) and PSN-19W (**b**) towards *C. elegans* exposed for 48 h with feeding. The asterisks indicate significance after filtration compared to the respective unfiltered control at *p* < 0.05.

Figure 8 illustrates reproductive toxicity of pH-adjusted MHRW solutions before and after filtration through PSN-19/PSN-19W. Figure 8a depicts a statistically significant increase in toxicity at pH 7 following filtration through PSN-19. Meanwhile in Figure 8b, this increase is not seen, likely due to annealing limiting solvent leaching. The lower toxicity observed in PSN-19W (annealed) membranes relative to PSN-19 (unannealed) under varied pH provides support for the annealing process minimizing NMP release during filtration.

Toxicity mechanisms related to solution filtration through PSN-19W is likely due to NMP utilized in membrane fabrication. NMP has a 5-membered ring structure with a heteroatom, and a methyl group [43]. Two potential toxicity mechanisms are due to NMP oxidation products formed (N-methylsuccinimide, 2-pyrrolidone, and succinimide. These have a Biochemical Oxygen Demand ratio (BOD_5_) and a Theoretical Oxygen Demand ratio (ThOD_5_). Prior studies highlight that NMP has (mg/L) a BOD_5_ of 728, ThOD_5_ of 1.94, and a BOD_5_/ThOD_5_ 0.38, while N-methylsuccinimide has 96, 1.41, and 0.01 respectively. 2-pyrrolidone has 452, 1.69, and 0.28, and succinimide has 537, 1.13, 0.48 [44]. The BOD_5_/ThOD_5_ ratio indicates biodegradability. If high, there is greater biological growth and decreased toxicity. The succinimide product is biodegradable and nontoxic, meanwhile methylsuccinmide had a low ratio so it is less biodegradable or more toxic. N-methylsuccinimide had the highest toxicity, followed by NMP (parent compound), and then 2-pyrrolidone and succinimide. Toxicity of solutions with dissolved NMP arise from interactions between the NMP, and the acid or base solution utilized for pH titration of the MHRW. If the oxidation is not carried out fully, the water’s toxicity is increased. Solutions become more toxic once oxidation starts, however once N-methylsuccinimide (primary toxicity mechanism) has been produced, toxicity decreases until stabilizing at hour 24. Reproduction exposures take place for 48 h at neutral pH, whereas mortality exposures take place for 24 h at a larger pH range allowing for greater interactions with oxidation, leading to increased apparent toxicity compared to reproduction exposures [44]. Cation and anion measurements in the permeates were conducted to rule out (other than NMP) potential causes that could have explained the increased toxicity following filtration through the membranes. The intent was to test whether the observed increase in *C. elegans* toxicity after filtration through PSN-19 (unannealed) membranes could have been explained by cation or anion (salt) removal from Moderately Hard Reconstituted Water (MHRW) during filtration. If MHRW, an optimal media for *C. elegans*, was depleted due to salt exclusion during filtration, this could have adversely affected nematode survival and reproduction. However, since no significant changes in anion and cation concentrations post filtration were detected, this potential cause for the higher toxicity post filtration was not confirmed.

#### 3.4.4. Total Nitrogen (TN) Concentrations After Filtration

Variability was observed for total Nitrogen concentrations after filtration at varying pH. However, at pH 7 (where we had observed a significant increase in toxicity) there was a consistent increase in total Nitrogen concentrations after filtration through PSN-19. Data for TN for PSN-19 and PSN-19W are provided in Table S3. The increased TN is likely attributed to solvent, such as NMP, or contaminants present during membrane fabrication that were trapped in pores being released following filtration through PSN-19.

## 4. Investigation of Membrane Performance 

**Figure 9:**
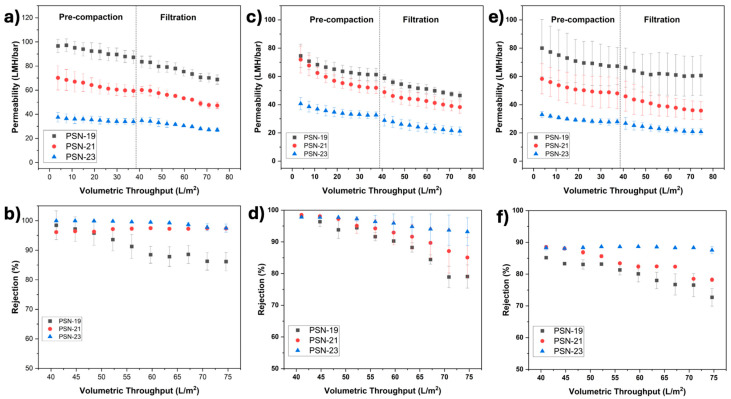
Membrane performance studies (**a**) permeability and (**b**) rejection of MB, (**c**) permeability and (**d**) rejection of CV, (**e**) permeability and (**f**) rejection of CR using PSN-19, PSN-21, and PSN-23 membranes.

﻿ The filtration performance of PSf-SPEEK membranes was evaluated using low molecular weight organic dyes in the water using a dead-end filtration cell. To distinguish the interaction of charge and size of the solute with membranes, cationic dyes (MB and CV) and anionic dyes (CR and AO2) were selected. Permeability and rejection results for MB using PSN-19, PSN-21 and PSN-23 membranes are shown in Figure 9a,b. As previously noted, increasing the polymer concentration would suggest a difference in membrane performance due to membrane properties differences. By conclusion of the pre-compaction of PSN-19 indicated its permeability of 68.8 LMH/bar and rejection of 86%. PSN-21 indicated lower permeability than PSN-19, but rejection increased to 97%. This suggests that MB rejection follows an antagonistic pattern to permeability. As polymer concentration of dope solution in PSN-23 increased, the permeability reduced further to 27.0 LMH/bar, and removal of MB increased to 98%. Figure 9b emphasizes that PSN-19 membranes exhibited rejection rate of 98% that decreased as volumetric throughput increased, however it remained stable as filtration continued for PSN-23. The increase in dope solution concentration to 23% led the membrane to exhibit a more restricted pore flow which worked synergistically with electrostatic charge attraction. This contributed to enhanced removal of MB by improving both charge-based and pore flow-dependent rejection mechanisms. This suggested that optimizing membrane composition enhances permeability and solute rejection. MB was used to further examine the filtration performance for pristine PSf membranes, average permeabilities, and rejections using PN-17 and PN-19, and PSf-SPEEK membranes PSN-17, PSN-19, PSN-21, and PSN-23 are shown in Figure S7, and discussed in S.5.4. The filtration results of pristine PSf membranes (PN-17 and PN-19), and PSN-17 with MB are shown in Figure S7. The permeabilities of PN-17 and PN-19 were significantly lower than PSN-17 and PSN-19 membranes. These elevated permeabilities are due to strong affinity of water for SPEEK due to functionalization of membranes with sulfonic acid groups. The incorporation of sulfonic acid groups to the backbone of the polymer structure in PSN-17, PSN-19, PSN-21, and PSN-23 led to increases in hydrophilicity, shown in Figure 9. Therefore, SPEEK incorporation provided a mass transport channel for water permeation which significantly improved permeability, and probable negative charge on the surface of membranes improved the rejection performance. Another cationic dye, CV, shown in Figure 9c,d, exhibited similar performance to MB. High rejection results for cationic dyes, MB and CV, indicates the presence of negative charges on the membrane surface.

CR, an anionic dye with relatively larger molecular weight, was also tested. Permeability and rejection results using PSN-19, PSN-21 and PSN-23 membranes are shown in Figure 9e,f. Permeability decreased and rejection increased as a function of volumetric throughput as polymer concentration increased. Minimum PSN-19 CR rejection was 75%, while in PSN-23 a rejection of 88% was observed. Figure 9f depicts that CR rejection in PSN-23 remained stable as filtration continued. This can be attributed to the larger CR molecular size, emphasizing that size exclusion became dominant factor in CR rejection at higher polymer concentration due to denser surface. Also, since the membrane is negatively charged, electrostatic repulsion may aid in achieving rejections to those for CR. It is observed that lower polymer concentrations, PSN-19 and PSN-21, showed decreased rejection as filtration progressed. The decline in rejection over time may happen due to charge shielding effect due to probable dye accumulation on the membrane surface. The structure of CR contains hydrogen bond donor and acceptor sites, their adsorption via hydrogen bonding with membrane surface can partially screen the membrane’s negative charge imparted by sulfonic acid groups [45]. This may weaken electrostatic repulsion, reducing the rejection efficiency as filtration progresses. The smaller anionic dye, AO2, had the lowest rejection rates—18% for PSN-19 and 38% for PSN-23, shown in Figure S8. Compared with the large CR, the smaller molecular size of AO2 allowed it to pass through the membrane’s pores and the charge on the membrane did not exert a strong influence on the smaller molecules. This suggests, membrane performance is subject to solute type and has broad application prospects for different type of solute rejection based on its characteristics as shown in Table 2.

The literature suggests that dye filtration performance with membranes exhibits sensitivity to factors including dye structure, and ionic content which cannot be explained by a single mechanism [46,47]. Table 3 shows the comparative study of membrane prepared in this work to other reported polymeric membrane performance in literature which follows different mechanisms. The data suggest pore size contributes to solute rejection, but the mechanism driving separation can be electrostatic interactions, both electrostatic attractions or repulsions, between the solute and the membrane. These attractive and repulsive interactions are influenced by the electrostatic environment between the membrane and the solute which affects overall effectiveness and separation mechanisms [48,49].

### 4.1. Selective Filtration Using Binary Dye Mixtures

**﻿ ** Considering the separation behavior of individual dyes, binary dye mixtures were studied to investigate how their interactions impact separation performance. The results demonstrated that despite the high solubility of organic dyes, selective separation was achievable. The selective filtration performance of the PSf-SPEEK nanofiltration membrane, PSN-19, was assessed using a binary dye mixture containing cationic dye (MB) and anionic dye (AO2). In the MB/AO2 binary system, the UV-vis spectrum of the binary dye solution showed two distinct adsorption peaks at 483 and 663 nm, as shown in Figure S9. The intensities of characteristic absorption peaks of individual dyes were used to calculate the rejection.

**Figure 10:**
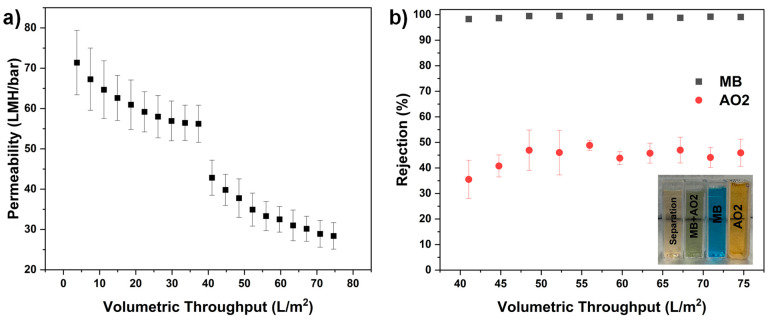
Filtration performance study of 50:50 mixture of Methylene Blue (MB) and Acid Orange 2 (AO2) as model dyes. (**a**) Permeability, (**b**) rejection using PSN-19 membrane.

Figure 9 illustrates that PSN membranes exhibit higher rejection for cationic dyes including MB than anionic dyes based on electrostatic effect and size exclusion. For the mixture of MB and AO2, PSN-19 showed selective behavior between MB and AO2. Due to the membrane’s negative surface charge, MB, a cationic dye, exhibited ~99% rejection throughout the filtration process. The rejection is attributed to electrostatic attraction and subsequent adsorption on both the membrane surface and within the pore structure. Meanwhile, AO2, an anionic dye, initially showed 34% rejection which gradually increased over time. MB adsorption on the membrane surface could partially neutralize the membrane’s negative charge over time which indirectly impacts AO2 rejection by increasing effective dye blocking due to fouling and charge modification effects over time. In Figure 10a, the observed decrease in permeability as filtration progressed is attributed to the formation of micron-sized aggregates resulting from intermolecular interactions between oppositely charged components in the binary dye mixture. Specifically, the cationic MB interacts with the AO2, leading to the formation of micron-sized aggregates that may block the membrane pores, thereby increasing resistance to flow. This aggregate formation in the binary dye solution led to a sharper decline in permeability as compared to single dye solutions. Tran et al. studied a detailed mechanism of binary dyes which proposed the same mechanism during filtration of mixtures of different cationic and anionic dyes [58]. In addition to these aggregate formations, changes in the electrostatic double layer (EDL) around the membrane pores may further contribute to the increased flow resistance [46]. Permeability and rejection changes can be due to micron-sized aggregate formation due to intermolecular interactions between oppositely charged dyes. The findings indicate that if the selective removal of specific components is required, the membranes can be designed for separation based on electrostatic interactions and molecular size exclusion.

### 4.2. Annealed Membrane Performance

**Figure 11:**
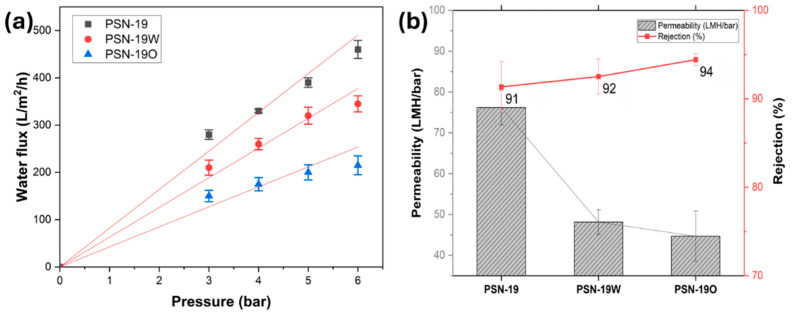
(**a**) Water flux studies of PSN-19, PSN-19W (water annealed), and PSN-19O (oven annealed) at varying pressures. (**b**) Permeability and rejection plots for PSN-19, PSN-19W, and PSN-19O membranes with MB dye.

Annealing effects on membrane performance were assessed using a PSN-19 membrane. Annealing is an effective methodology for shrinking the voids between intra-molecular chain segments due to efficient chain packing in amorphous or semicrystalline polymers [41]. NIPS involves the rapid coagulation of the polymer leading to a highly dispersed structure that is not thermodynamically stable. Thermal annealing may enhance the molecular motion of polymer chain segments and cause morphological changes in the polymer network [59,60]. Water flux performance of the membranes was tested at varying transmembrane pressures to understand annealing effects on permeability, shown in Figure 11a. Ideally, water flux follows a linear relationship with applied pressure. A slight non-linearity can be attributed to membrane compaction and structural adjustments. According to the literature, these effects are more pronounced at lower pressures and may cause deviations from the expected linear trend in water flux versus pressure [61,62]. PSN-19O and PSN-19W exhibited pure water flux reduction compared to PSN-19, with PSN-19O demonstrating a more prominent decrease. Even though morphological changes may be limited, the porosity changes illustrate that annealed membranes became structurally compact due to applied thermal treatment allowing polymeric chains to organize into configurations that are more thermodynamically stable. Thermal treatment may have removed residual solvents that enhanced overall packing of polymer chains in the membrane matrix. This overall compaction reduced the free volume available for water passage, lowering the flux [19]. Despite an overall water flux reduction after annealing, both sets of annealed membranes had increased water flux as transmembrane pressure increased. This was expected, due to the driving force for water transport across the membrane increasing at higher pressures. Rejection performance was evaluated using MB as a model dye, shown in 11b. Rejection performance of annealed membranes improved although not significantly. Rejection rates of MB increased from 91.35% in PSN-19 to 92.51% in PSN-19W and 94.42% in PSN-19O membranes. The slightly increased performance might be due to increased resistance to dye permeation as a result of more densely packed polymer matrixes caused by shrinkage of voids between nodule aggregates and the inter-chain spaces within molecular structures [42,63]. Few studies have assessed annealing effects on membrane performance and since different polymers behave differently to annealing, further research across a range of polymer types is required to reach definitive conclusions about its impact on membrane properties.

## 5. Conclusions

The nonsolvent induced phase separation (NIPS) fabrication, characterization and toxicological assessment of PSf/SPEEK membranes with varying polymer concentrations were investigated here. The sulfonation of PEEK enabled SPEEK’s solubility in NMP, while its incorporation in a polysulfone polymer matrix increased membrane hydrophilicity. Furthermore, the addition of SPEEK increased porosity, which compromised mechanical strength. However, as the SPEEK polymer concentration increased, the mechanical strength improved due to the higher density of polymer chains. The membranes exhibited a finger-like pore structure, which is preferred for water applications, as it combines a dense skin layer with low resistance bottom layer. Post-treatment via annealing reduced water flux without significantly changing dye rejection but notably decreased membrane toxicity at pH 7. Prior to annealing, permeates filtered through unannealed membranes at pH 7 exhibited increased toxicity compared to unfiltered pH 7 solutions. Post annealing, permeate toxicity following membrane filtration was not significantly different from unfiltered solutions of the same pH. Membrane annealing helps reduce the presence of membrane fabrication solvents (e.g., NMP) in permeates which are toxic towards the model organism *C. elegans*. Performance testing revealed that rejection mechanisms varied with dye type: larger anionic dyes showed size exclusion, while cationic dyes demonstrated strong rejection through synergistic size exclusion and electrostatic attraction which facilitated subsequent adsorption. So, these results suggest that membranes exhibit different filtration mechanisms depending on solute type. Overall, these findings provide insights into SbD approach to optimize membrane performance while minimizing environmental risks.

## Figures and Tables

**Table 1:** Mean total porosity measured by gas pycnometer, and mechanical strength using tensile strength analyzer of all synthesized membranes.

Membrane	Porosity (%)	Tensile Strength (MPa)
PN-17	63.1 ± 3.43	4.40 ± 0.14
PN-19	64.9 ± 3.75	5.28 ± 0.19
PSN-17	86.4 ± 3.6	3.65 ± 0.14
PSN-19	82.1 ± 1.56	3.86 ± 0.45
PSN-21	80.13 ± 1.79	5.27 ± 0.12
PSN-23	78.5 ± 2.14	5.71 ± 0.21
PSN-19W	79.4 ± 3.21	4.84 ± 0.25
PSN-19O	78.9 ± 0.89	4.17 ± 0.60

**Table 2:** Performance comparison based on dye properties.

Model Dye	Molecular Weight (g/mol)	Ionic Nature	Observed Rejection	Observed Behavior Based on Performance
MB	319.85	Cationic	99%	High rejection due to size exclusion and strong electrostatic attraction between the cationic MB and the negatively charged membrane surface, combined with moderate molecular size.
CR	696.7	Anionic	86%	Good rejection since CR is a large, anionic dye. The electrostatic interactions, and size exclusion helped achieve relatively high rejection.
CV	407.99	Cationic	95%	High rejection due to electrostatic attraction between the cationic nature and the membrane’s negative charge. CV’s moderate size contributed to efficient rejection.
AO2	350.22	Anionic	38%	Low rejection due to both smaller molecular size and electrostatic repulsion between the anionic AO2 and the negatively charged membrane surface. The smaller size of AO2 resulted in low retention.

**Table 3:** Comparative performance studies with polymeric membranes using different dyes.

Membrane	Process	Flux (LMH)	Pressure (Bar)	Feed	% Removal	Suggested Mechanism	Ref.
Polyvinylidene fluoride/chitosan/dopamine membranes	UF	116–201	1	OG	96.8% (MB) 92.7% (OG)	Electrostatic attraction	[50]
PSf/sulfonated-TiO_2_	NF	6.50	6	MB	90.4%	Electrostatic attraction/adsorption	[51]
UiO-66-NH2/(GO) on polyurethane composite membranes	MF	-	-	MB, CR	95% (MB), 90% (CR)	Electrostatic attraction and hydrogen bonding	[45]
Cellulose acetate/metal-organic framework adsorptive membrane	UF	76.03	1	MB	98.2%	Electrostatic attraction	[52]
Hydrolyzed PAN-ETA	UF	50–53	2	MB, CV	96%	Electrostatic interaction and adsorption	[46]
Polyethersulfone nanofibrous membrane	MF	-	-	CR, and Cd	-	Electrostatic attraction and adsorption	[53]
Polythyleneimine-modified positive charged TFC NF membrane	NF	34–38	10	TO (+), VB (−), SO (−), NR (+)	>98%	Pore size, electrostatic interactions	[54]
Cellulose acetate (CA)-based membranes by phase inversion and electrospinning	MF	-	-	MB and CR	31 to 70% (MB) and <10% (CR)	Electrostatic attraction	[55]
PES/Fe_3_O_4_@SiO_2_	NF	70.6	4	MB	98	Donnan exclusion and adsorption	[56]
PVDF/Hydroxyapatite Nanoparticles UF Membrane	MF	400	1	CR	88%	Electrostatic attraction, Lewis interaction	[57]
PSf/SPEEK	UF	90	4.13	MB CV CV MB + AO2	99% (MB) 97% (CV) 87% (CR) MB (99%)	Electrostatic interactions, Size exclusion	This work

Abbreviations in table Orange G (OG), Tropaeolin O (TO), Victoria Blue (VB), Semixylenol Orange (SO), Neutral Red (NR), Microfiltration (MF), Ultrafiltration (UF), Nanofiltration (NF).

## Data Availability

The data presented in this study are available upon request from the corresponding author.

## Supplementary Materials

The following supporting information can be downloaded at: https://www.mdpi.com/article/10.3390/membranes15030087/s1, Figure S1: Schematic diagram of the process used to synthesize SPEEK; Figure S2: Cloud point measurements obtained by titration method, shown in ternary diagram of polymer (PSf and PSf-SPEEK), NMP (solvent) and water (non-solvent) system, with blue and green line showing the precipitation path; Figure S3: Viscosity of dope solutions prepared at different concentrations using PSf and PSf-SPEEK, measured at different shear rates in steady-shear flow, ranging from 0 to 90 s^−1^; Figure S4: FTIR spectra showing the functional peaks comparison of fabricated membranes PN-19, PSN-19, and PSN-23; Figure S5:XPS characterization of membrane chemistry. XPS spectra of (a) PN-19 (b) PSN-19, and (c) PSN-19O; Figure S6: SEM cross-sectional and surface images of (a) PSN-17 and (b) PSN-23 membranes; Figure S7: Average Permeability and rejection plot for MB dye with PN-17, PN-19, PSN-17, PSN-19, PSN-21, and PSN-23; Figure S8: Average Permeability (left) and rejection data (right) of PSN-19 and PSN-23 membranes with MB, CR, CV, and AO2; Figure S9: UV-Vis spectra of Mixed dyes (MB + AO2) plotted after every 5 mL of filtration; Table S1: Key Properties of dyes (Congo Red, Methylene Blue, Crystal Violet, and Acid Orange 2) relevant to membrane filtration performance; Table S2: Elemental composition of C1s, O2s, S2p of PN-19, PSN-19, PSN-19O and PSN-19 (etched at 300 s) using XPS; Table S3 TN (mg/L) prior to and following filtration of MHRW at pH 7 through PSN-19 and PSN19W.
