# Supplementary material for: Characterization, Performance, and Toxicological Assessment of Polysulfone-Sulfonated Polyether Ether Ketone Membranes for Water Separation Applications
Source: Membranes (Basel). 2025 Mar 8;15(3):87. doi: 10.3390/membranes15030087 (PMC11943734; doi:10.3390/membranes15030087)
Supplement: Supplementary file 1 [file membranes-15-00087-s001.zip › membranes-3482162-supplementary.pdf]

Supplementary Information for
Characterization, Performance, and Toxicological Assessment of Polysulfone-Sulfonated Polyether Ether Ketone Membranes for Water Separation Applications

Muhammad Usman Yousaf [1,†], Lucca Madeo Cortarelli [2,†], Nerissa I. Jebet [3], Jason M. Unrine [2,4], Nirupam Aich [5], Olga V. Tsyusko [2,*], Isabel C. Escobar [1,*]

1 Department of Chemical and Materials Engineering, Stanley and Karen Pigman College of Engineering, University of Kentucky, Lexington, KY 40506, USA; usman.yousaf@uky.edu
2 Department of Plant and Soil Sciences, Martin-Gatton College of Agriculture, Food and Environment, University of Kentucky, Lexington, KY 40546, USA; lucca.cortarelli@uky.edu (L.M.C.); jason.unrine@uky.edu (J.M.U.)
3 Department of Health and Clinical Sciences, College of Health Sciences, University of Kentucky, Lexington, KY 40506, USA; njki227@uky.edu (N.I.J.)
4 Kentucky Water Research Institute, University of Kentucky, Lexington, KY 40506, USA
5 Department of Civil and Environmental Engineering, College of Engineering, University of Nebraska–Lincoln, Lincoln, NE 68588, USA; nirupam.aich@unl.edu
* Correspondence: olga.tsyusko@uky.edu (O.V.T.); isabel.escobar@uky.edu (I.C.E.)
† These authors contributed equally to this work.

S.1 Introduction

Literature on annealing: Membranes may leach solvent trapped within membrane pores during filtration, leading to solvent release into effluents. To address this, after fabrication, polymeric membranes undergo thermal annealing process, which allows any trapped solvent to be released due to changes in membrane structure and ion conductivity [1]. Annealing improves film morphology and enhances interchain interaction of polymeric chains leading to more compact membranes, especially in polymer blends, conjugated polymers, and ion-conducting polymers [2]. PSf-ZNO nanohybrid blended membranes fabricated by NIPS have been investigated [3]. It is observed that the polymer packing density, mechanical strength, and surface hydrophilicity were affected by changes in structure and membrane performance due to differences in thermal annealing duration [4]. Di Vona et al. studied the annealing effects on SPEEK membranes used for proton exchange in fuel cell at temperatures between 120 and 160°C. Thermal annealing promoted cross-linking through SO_2 bridges between polymer chains which significantly reduced water uptake, minimized swelling, and enhanced mechanical properties [5]. In a study by Jung et al., thermal annealing of polyamide membranes in hot water enhanced mechanical strength and salt rejection while reducing pore size, which decreased water flux [6]. Before annealing, membranes may have trapped solvent in their pores. However, after annealing, pore size reduction and increased salt rejection simultaneously likely decreases amount of trapped NMP solvent onto the annealing solution, while also increasing selectivity of filtered solutions which decrease the toxicity of filtered permeates. Thus, annealing proves essential for tailored membrane performance in specific applications while simultaneously reducing toxicity of solutions filtered through them. Polymers lead to differential performance under thermal treatment, which necessitates an investigation of annealing effects on performance, structure, and toxicity of filtered solutions [7].

A Safe-by-Design (SbD) approach was adopted to assess potential toxicity at all stages of NF membrane development. Potential origins of toxicity in membrane fabrication and usage were identified, particularly related to solvents such as NMP, which could leach from membranes into permeates. Toxicity was assessed using the model nematode, *Caenorhabditis elegans*, chosen for its high reproductive capacity, short generation time, and fully sequenced and annotated genome [8]. *Caenorhabditis elegans* are essential to meet the National Institutes of Health's (NIH) has goals for refining, reducing, and replacing vertebrate animal testing in toxicology. Toxicity ranking screens have shown high predictive potential for rats and mice, and various modes of toxic action conservation have been identified between mammals and *C. elegans* [9]. Additionally, *C. elegans* shares 60-80% of protein-coding genes that are homologous to humans [10] underscoring its significance as a model organism for toxicity testing in SbD product development.

S.2 Materials

For Polymer synthesis and membrane fabrication Polyether ether ketone (PEEK) (Polysciences Inc., Warrington, PA, USA), Sulfuric acid (H_2SO_4) for PEEK sulfonation (VWR international, Solon, OH, USA), and Dimethyl sulfoxide-d6 for NMR spectroscopy (Millipore Sigma, Burlington, MA) were utilized. Membrane synthesis and performance studies required usage of polysulfone (PSf) with molecular weights (mw) of 35,000 as base polymer (Sigma Aldrich, St. Louis, MI, USA). Additionally, N-Methyl-2-pyrrolidone (NMP) was selected as the solvent (VWR International, Solon, OH, USA). Organic dyes selected were Methylene Blue, Congo Red, Crystal Violet and Acid Orange-2, and salts including $CaCl_2$ and Na_2SO_4 (Sigma Aldrich, St. Louis, MI, USA).

For toxicity assays, Moderately Hard Reconstituted Water (MHRW), a standard EPA toxicity medium, was prepared using the following concentrations: 0.00114 M NaHCO3, 0.000348 M CaSO4·2H2O, 0.000497 M MgSO4·7H2O, and 0.000054 M KCl [[11] (U.S. EPA, 2002). pH was then adjusted through titration with sulfuric acid or sodium hydroxide. All toxicity studies were conducted in MHRW.

S.3 SPEEK synthesis and Membrane preparation

S.3.1 Synthesis of Sulfonated PEEK (SPEEK)

SPEEK sulfonation techniques were previously researched, and procedure adapted from our previous work [12,13]. PEEK sulfonation for SPEEK production involves dissolution and sulfonation in concentrated sulfuric acid. PEEK (50 g) was dissolved in sulfuric acid at 60°C to form a dark brown viscous solution. The solution was kept under constant agitation for 8 hours to allow sulfonation to occur. The degree of sulfonation (DS) is highly dependent on time and temperature of sulfonation reaction. The reaction was terminated in ice-cold water bath where phase inversion of the viscous solution to a solid occurred. The precipitant was filtered and washed repeatedly with DI water until a neutral pH was achieved. The resulting polymer was dried overnight in a vacuum oven at 80°C for membrane fabrication. The schematic of synthesis

procedure is shown in fig. S1. PEEK sulfonation increases its hydrophilicity and improves its solubility in solvents.[12,14,15] The solubility of SPEEK depends on its DS as reported in several studies. 30% DS allows it to dissolve in DMF, DMSO, and NMP when heated, while at 40–70% DS, it is soluble in these solvents at room temperature. DS greater than 70% allows for dissolution in methanol and at DS of 100% it is soluble in hot water [12,16,17]. Therefore, DS parameters are essential to utilize PEEK for membrane fabrication.

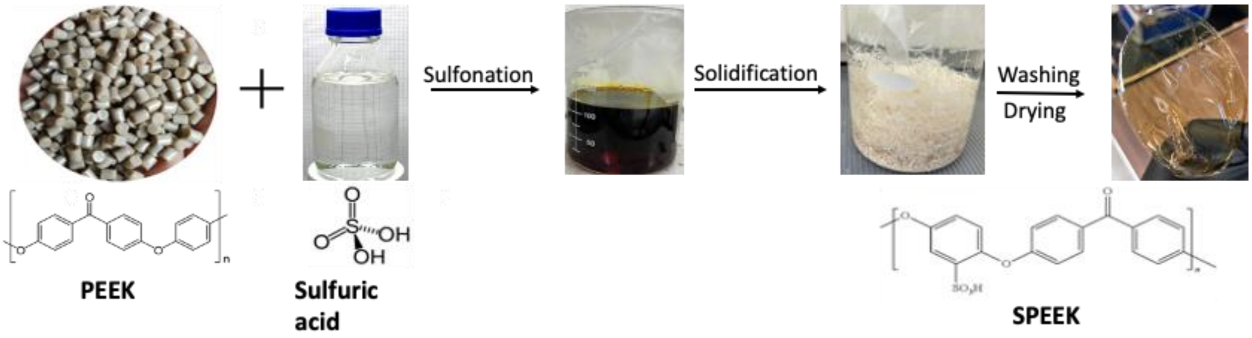

Figure S1. Schematic diagram of the process used to synthesize SPEEK.

S.3.2 Preparation of dope solution

Blended PSf and SPEEK were utilized for fabrication of integrally skinned asymmetric membranes using NIPS casting. Dope solution polymer concentration varied at 17, 19, 21, 23% wt.%, while SPEEK concentration was maintained at 5%. Five weight percent SPEEK along with varying weight percentages of PSf were dissolved in NMP solvent at room temperature and stirred continuously to ensure homogenized dissolution. Afterwards, PSf was added to the solution and stirred to synthesize a PSf-SPEEK homogenized blend. Solutions were sonicated for an hour at room temperature and degasified for 2 hours to remove air bubbles prior to membrane fabrication.

S.3.3 Preparation of PSf/SPEEK membranes fabrication

The membranes were fabricated using the NIPS method. The dope solution was cast onto a glass plate using a doctor blade (Micrometer Adjustable Film Applicator – 250 mm, MTI Corp, Richmond, CA, USA) at a coating gap of 0.2 mm. After approximately 10 s of evaporation time, the cast film was immersed into a non-solvent bath (DI water) to induce phase separation. The immersion time was allowed for the formation of asymmetry in the membrane structure. Following phase separation, membranes were removed from the bath, rinsed with DI water to remove residual solvents, and stored in a water bath for at least 24 h at room temperature prior to testing. To fabricate the membranes under SbD approach, post-treatment annealing was performed using two different strategies: oven annealing and water annealing. For water annealing, membranes were immersed in deionized water at 80°C for 45 minutes. For oven annealing, membrane (PSN-19) was heated at 120°C for 1 minute. The annealing temperature was

maintained below the glass transition temperature of the polymers to prevent structural degradation.

Membranes with 17% and 19% PSf were labeled as PN-17 and PN-19, respectively. Following SPEEK addition, membranes were designated as PSN-17, PSN-19, PSN-21, and PSN-23. The oven-annealed and water-annealed membranes were labeled as PSN-19O and PSN-19W, respectively.

S.4 Characterization, Toxicology Assessment, and Experimental Performance

S.4.1 Hydrogen Nuclear Magnetic Resonance (H-NMR) Analysis

H-NMR spectroscopy was performed with Bruker NMR (Billerica, MA, USA) at a frequency of 400 MHz to determine the degree of sulfonation (DS) in SPEEK. Prior to testing, SPEEK was dissolved in deuterated dimethyl sulfoxide (DMSO-d_6) to investigate the proton chemical shifts in the polymer backbone. The peaks corresponding to sulfonic acid groups were identified, and the DS was calculated by integrating these peaks (in Bruker Topspin software) relative to the aromatic protons, given by equation S1 [12,18,19].

$$\frac{DS}{12-2DS} = \frac{S_{H13}}{S_{H(1,2,3,4,5,6,7,8,9,10,11,12,14,15)}} \quad \text{(S1)}$$

S.4.2 Differential Scanning Calorimetry (DSC)

DSC was performed using ThermoFisher Scientific DSC (Hillsboro, OR, USA) to investigate the glass transition temperature (T_g) of the synthesized SPEEK. The sample was dried prior to analysis, and heating at ramp rate of 10°C/min supplied up to 230°C.

S.4.3 Cloud point determination

For the polymer/solvent/non-solvent, cloud points were experimentally measured using titration with water as non-solvent. Six dope solutions of PSF and six PSF/SPEEK blends, with the same ratio used in membrane fabrication, were prepared at polymer concentrations of 15%, 17%, 19%, 21%, 23%, and 25%. At room temperature, 5 ml DI water was incrementally added using a micropipette until turbidity stabilized after 24 hours of stirring. If turbidity remained, the polymer volume fraction was considered as the cloud point. The amount of non-solvent required to induce polymer precipitation provides an estimate of the thermodynamic stability [20,21].

S.4.4 Dope solution viscosity

Dope solution viscosity was analyzed using a digital rheometer (AG-G2, TAinstrument, DE, USA) at a steady shear flow rate ranging from 0 to 90 s^{-1}. Experiments were performed at room temperature and solutions were completely homogenized prior to measurements.

S.4.5 Fourier Transform Infrared Spectroscopy (FTIR)

The surfaces of the membranes were analyzed using FTIR measurements to investigate the surface chemical functionality of the synthesized membranes. Spectra were recorded using a NEXUS 470/670/870, 110 W, 5 V/12 V ESD (Thermo Nicolet, Madison, WI, USA) over the range of 4000 to 500 cm^{-1}. This allowed for the identification of specific peaks corresponding to PSf and SPEEK's chemical structure that is essential for understanding the surface chemistry of the membrane. Membranes were air-dried prior to analysis.

S.4.6 Scanning Electron Microscopy (SEM)

SEM imaging was performed using FEI Quanta 250 scanning electron microscopy (SEM, ThermoFisher Scientific, Hillsboro, OR, USA) to examine the surface and cross-sectional morphology of the membranes. The samples were dried prior to imaging and sputter-coated with platinum. For cross sectional analysis, high-resolution images are captured after cryo-snapping in liquid nitrogen. The images were taken in a high vacuum (10^{-6} mbar) in secondary electron mode.

S.4.7 X-ray Photoelectron Spectroscopy (XPS)

XPS spectroscopic measurements were taken to analyze the surface elemental composition of the membrane, with a focus on carbon, oxygen, and sulfur using a Thermo Scientific K-Alpha XPS (Waltham, MA, USA). Moreover, depth-profile measurements were carried out to determine any elemental composition differences between surface and inside the membranes, and to investigate the SO_4^{3-} functionalization on the surface and inside the membrane structure. The membranes were scanned over a range of 0-1400eV.

S.4.8 Hydrophilicity

The relative hydrophilicity and hydrophobicity of the membranes were evaluated via water contact angle measurements obtained using a drop shape analyzer Kruss DSA100 (Matthews, NC, USA) in a sessile drop mode. At least three independent membrane samples were air-dried before measurements. The membranes were cut into specific dimensions, a 5 μl deionized water droplet was deposited on the surface, and measurements were taken within 10 s.

S.4.9 Porosity

The mean porosity of the membranes was determined using a gas pycnometer by Anton Parr Ultrapyc (Graz, Austria). Multiple membrane coupons with radius of 1.27 cm were inserted into pycnometer with set helium gas pressure of 4 psi. This measured the volume of gas displaced by the sample based on changing the pressure of the gas. The porosity was calculated by comparing the known density of the membrane and density measured through a pycnometer, using equation S2. Each measurement was performed 10 times by pycnometer and values were reported with deviation from the instrument.

$$\text{Total porosity (\%)} = 1 - \frac{r_{pycnometer}}{r_{geometry}} \times 100\% \qquad (S2)$$

where $r_{pycnometer}$ represents the density of the coupons determined by the gas pycnometer and $r_{geometry}$ represents the density of the coupons based on the weight and dimensions.

S.4.10 Mechanical properties

The mechanical strength of membranes was tested using Instron Universal Testing System (Norwood, MA, USA). Samples were prepared by cutting them into a standardized dog-bone shape, which helps to concentrate stress in the central region and reduce edge effects during testing. Each membrane sample was subjected to tensile stress until failure to determine the ultimate tensile strength (in MPa). Three specimens per membrane type were tested, and the average value was reported.

S.4.11 Toxicity assays

An N2 strain of nematodes from the *Caenorhabditis* Genetic Center (CGC, Minneapolis, MN, USA), which has been widely utilized in laboratory studies [22], was utilized. Unless otherwise specified, all nematodes were fed *Escherichia coli* OP50, a slow-growing, non-pathogenic strain that cannot synthesize uracil and is not able to form a biofilm, making it suitable for nematode feeding [23,24]. OP50 was cultured prior to each exposure, and dead bacteria were confirmed as a viable food source for the nematodes [24,25].

S.4.12 C. elegans maintenance, age-synchronization, and acclimation

Established protocols were followed for all procedures [11,26-28]. Nematodes were transferred weekly onto separate K-agar plates containing a lawn of *E. coli* OP50 for 72 h. Age synchronization was achieved using a NaClO/NaOH solution to isolate eggs [24]. Nematodes were then acclimated to a low ionic strength MHRW.

S.4.13 Filtration of solutions at varying pHs through PSf/SPEEK membrane for toxicity assays

MHRW solutions adjusted to pH levels of 3, 5, 7, and 9 were used, as *C. elegans* is highly resistant to pH stress and thrives in a range from 3.13 to 11.33 [29]. Membranes were cut to an area of 14.6 cm^2 and placed in a 50 mL Amicon Stirred Cell. Deionized water was filtered through membranes at 60 psi for purging. Next, 30 mL of the titrated MHRW solution was filtered through the cell, with the permeates being refiltered five times. New membranes were utilized for each pH solution, and the same procedure was followed for both unannealed and annealed membranes.

S.4.14 C. elegans mortality assays

Following nematode hatching (~48 h), L3-stage nematodes were acclimated to MHRW and subsequently exposed to varying solution concentrations. A 24-well plate format was utilized, including a control with MHRW and a reference toxicant (Cadmium at 21 mg/L from Cadmium chloride) known to induce 50% lethality. Treatments consisted of both unfiltered and filtered (five times) permeate solutions. Approximately 240 nematodes were exposed for 24 hours, followed by lethality assessment through counting.

S.4.15 C. elegans reproduction assays

Following nematode hatching (~24 hours), L1-stage nematodes were acclimated to MHRW and then exposed to various solution concentrations with *E. coli* OP50 as food supply. A MHRW control and two treatments are utilized. Treatments included both unfiltered and filtered (five times) permeate solutions. Following ~48 hours, nematodes were individually transferred onto plates with *E. coli* OP50 and are then incubated for 48 hours. Afterwards, plates were stained with Rose Bengal and heated to 50°C. Plates were then counted.

S.4.16 Statistical analysis

All toxicity data were carefully checked for outliers using Grubb's/Extreme Standard Deviant (ESD) test. Shapiro-Wilks Normality Test was run on to test for normal distribution. For the normally distributed data, the Kruskal-Wallis tests followed by post-hoc Dunn's test were conducted to test whether treatment including annealing, pH, and filtration had an effect and for pairwise comparison within each treatment with their respective controls.

S.4.17 Membrane performance

A stirred dead-end filtration cell by Millipore® (Burlington, MA, USA) capable of operating at a maximum pressure allowance of 5.17 bar (75 psi) was used for measuring water permeability, and rejection performance of the membranes with different type of solutes. Pressurized nitrogen gas was used to provide the pressure required for water permeation, and the permeation time for a fixed volume of 5 mL was recorded to calculate the water flux. Prior to testing, membrane samples (effective area: 14.6 cm^2) were pre-compacted with DI water at a pressure of 4.14 bar (60 psi) to stabilize flux and minimize compaction effects.

Filtration experiments were performed using aqueous solutions of organic dyes with different molecular weights, charges, and hydrated radii including Congo Red (CR), Methylene Blue (MB), Crystal Violet (CV), and Acid Orange 2 (AO2). Each dye was prepared at a stock concentration of 10 ppm and stored in dark containers for further use. For binary dye mixture, the MB and AO2 solutions were mixed at 50:50 ratio. This information would help to understand how the membranes perform in filtering specific solutes, especially in water treatment applications. Filtration experiments were conducted at a constant pressure of 4.14 bar (60 psi). Dye rejection

and flux were measured. Salt rejection experiments were performed at 1 g/L $CaCl_2$ and Na_2SO_4 solution each. The Cary 60 UV-Vis spectrophotometer (Agilent, Santa Clara, CA) and a conductivity meter (SG23- FK2, Mettler Toledo, Columbus, OH) were used to analyze dye and salt concentrations, respectively. Permeate was collected in 5 mL intervals for subsequent analysis of rejections. The chemical structure and properties of the dyes are given in Table S1. Water permeance (A_w) and rejection (R%) of organic dyes and salt were calculated using equation S3 and S4 respectively.

$$A_w = \frac{V}{A \times \Delta t \times \Delta P} \qquad (S3)$$

$$R = (1 - \frac{C_p}{C_f}) \times 100\% \qquad (S4)$$

Where V (L) is the volume of the permeated water, A (m^2) is the effective membrane area, Δt is the permeation time (hr), ΔP (bar) is the pressure difference across the membrane, and Cp and Cf are the concentration of permeate and feed solutions, respectively.

Table S1. Key Properties of dyes (Congo Red, Methylene Blue, Crystal Violet, and Acid Orange 2) relevant to membrane filtration performance.

Dye	Chemical Structures	Molecular weight (g/mol)	Molecular size	Charge	Chemical structure	lmax (nm)	Ref.
Methylene blue (MB)	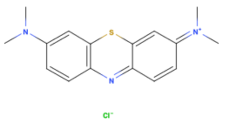	319.9	2.4 nm x 0.8 nm	Cationic	Thiazine dye	663	[30,31]
Congo Red (CR)	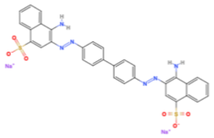	696.7	2.5 nm x 0.9 nm	Anionic	Azo dye	496	[30]
Crystal violet (CV)	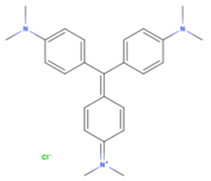	408.0	1.15 nm x 0.83 nm	Cationic	Triphenyl metane dye	590	[30,31]
Acid orange 2 (AO2)	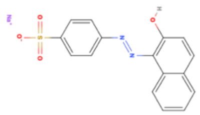	350.3	1.15 nm x 0.86 nm	Anionic	Azo dye	480	[32]

S.5 Results and Discussion

S.5.1 Dope Solution Behavior: Cloud Point and Viscosity

Phase separation processes including NIPS are challenging to monitor and analyze solely through experimental methods due to the speed of the procedure, dynamic variables, and intricate interdependencies. The process can partially be explained by assessing thermodynamics of the system through cloud point determination and kinetics through viscosity measurements.

The cloud points were determined to obtain a ternary phase diagram of polymer/solvent/nonsolvent systems. This was used to explore the thermodynamic behavior of the dope solution, determining the compositional path for membrane precipitation. The precipitation points of PSf and PSf-SPEEK solutions using water as a non-solvent is shown in fig S2. In a true ternary system, the cloud points coincide with the binodal curve, which represents the set of compositions where different phases coexist in thermodynamic equilibrium and it delineate regions of completely stable (single-phase) and meta- or unstable (phase-separated regions) [33]. SPEEK addition to the PSf matrix (PSf-SPEEK) in the casting solutions shifts the cloud point curve (shown in green), indicating that less water is required to induce precipitation. This suggests that the solutions become thermodynamically less stable with the inclusion of SPEEK. Cloud point determination reveals that this decreased stability arises from changes in the balance between enthalpic (interaction-based) and entropic (mixing-based) contributions to the free energy of the system [34,35]. A thermodynamically less stable polymer solution, such as those containing SPEEK, undergoes faster demixing when immersed in a nonsolvent due to enhanced thermodynamic immiscibility. This rapid phase separation can significantly alter the membrane characteristics which may affect its structure and performance.

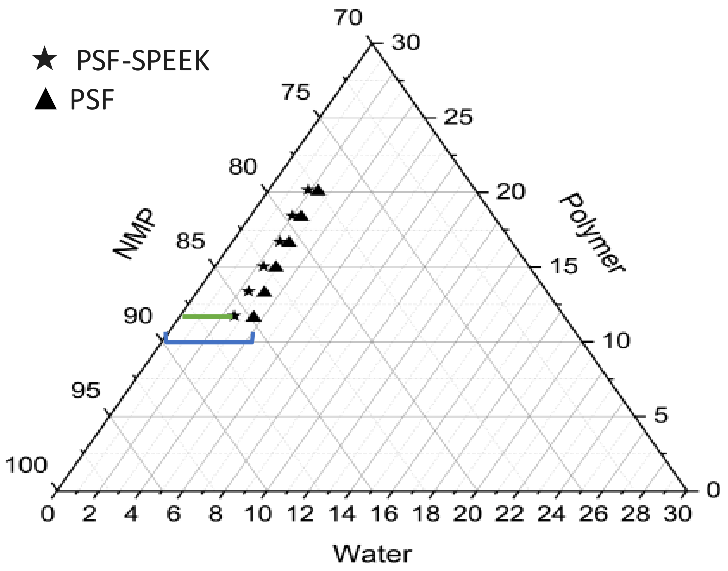

Figure S2. Cloud point measurements obtained by titration method, shown in ternary diagram of polymer (PSf and PSf-SPEEK), NMP (solvent) and water (non-solvent) system, with blue and green line showing the precipitation path.

Dope solution viscosity is a key parameter that impacts dissolution kinetics (during phase inversion) and resulting membrane structure and performance. Generally, polymer concentration increases solution viscosity due to increased polymer chain entanglement [36]. Fig S3 illustrates a similar trend, where PN solutions exhibit a viscosity increase from 0.39 Pa.s for PN-17 to 0.72 Pa.s for PN-19. Similarly, the PSN solutions, exhibit a similar trend which increase viscosity from 0.44 Pa.s (PSN-17) to 2.17 Pa.s (PSN-23) as polymer concentration increases. SPEEK-based membranes (PSN-17 and PSN-19) demonstrate slightly higher viscosities compared to their PSf counterparts (PN-17 and PN-19). This supports the observation that in addition to polymer dope concentration, molecular interactions can also contribute to increased viscosity. Kinetic aspects of the NIPS process are partially explained through viscosity. Increased viscosity of the dope solution causes the solvent and non-solvent exchange rate to decrease, leading to delayed demixing. On the other hand, lower viscosity correlates with rapid de-mixing during membrane fabrication [37]. Rapid demixing results in finger-like structures, while slower demixing produces sponge-like structures with thick skin layer [38,39].

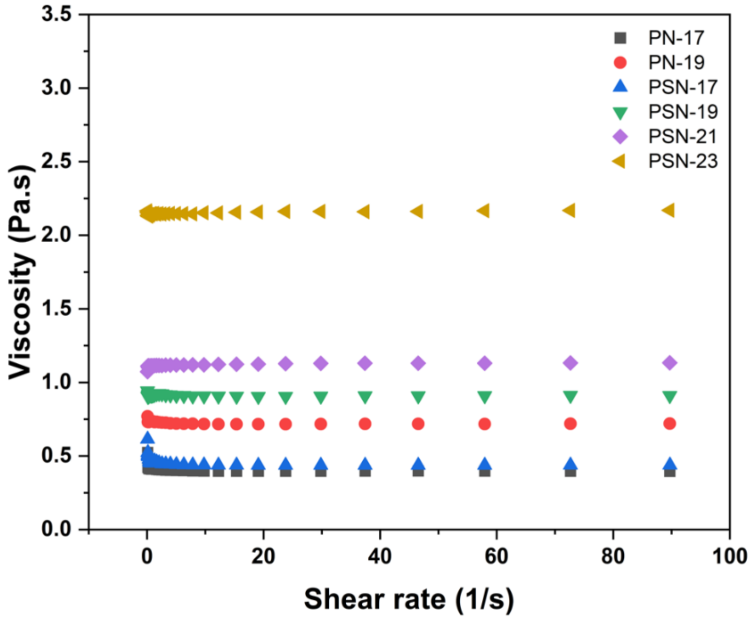

Figure S3. Viscosity of dope solutions prepared at different concentrations using PSf and PSf-SPEEK, measured at different shear rates in steady-shear flow, ranging from 0 to 90s^{-1}.

S.5.2 Membrane Characterizations

The FTIR spectra were used to confirm the surface functional groups of fabricated membranes. Fig S4 shows spectra of fabricated membranes blended with SPEEK. The PSf and SPEEK share some of the same functional groups in the polymeric structure. In addition to the distinctive bands of the PSf membrane, additional peaks (pointed by dotted lines) were observed at 1649 cm^{-1} and 1052 cm^{-1} in the spectrum of the PSf-SPEEK membrane. The peak at 1052 cm^{-1} corresponds to the presence of SO_4^{3-} groups, and 1649 cm^{-1} represents the C=O group. The appearance of new band confirms the presence of SPEEK in addition to PSf in membrane matrix.

XPS surface analyses of the pristine PSf membrane (PN-19), the PSf-SPEEK membrane (PSN-19), and the annealed PSF-SPEEK membrane (PSN-19O) are shown in Fig S5. The spectra show changes in the elemental composition and chemical environment upon blending and annealing. The elemental composition of C1s, O2s, S2p of PN-19, PSN-19 and PSN-19O are shown in table S2. In PN-19, the high atomic percentage of carbon (83.61%) is consistent with the PSf polymer backbone, while the presence of oxygen (14.02%) and sulfur (2.37%) correspond to ether and sulfonyl groups, respectively. Upon blending with SPEEK in PSN-19, a shift in the C1s peak to 285.02 eV, with a decrease in carbon content (81.55%) and an increase in oxygen (15.62%), shows the incorporation of SO_3H from SPEEK. The annealing process (PSN-19O) induced shifts in the binding energies and elemental percentages, with a slight reduction in carbon (80.77%) and increase in oxygen (16.18%), possibly due to cross-linking, or reorganization of the sulfonated domains, which may have enhanced the interaction between PSf and SPEEK during

annealing.[40] These changes show the chemical modifications are induced by incorporation of SPEEK and the thermal effects of annealing on the membrane's surface chemistry.

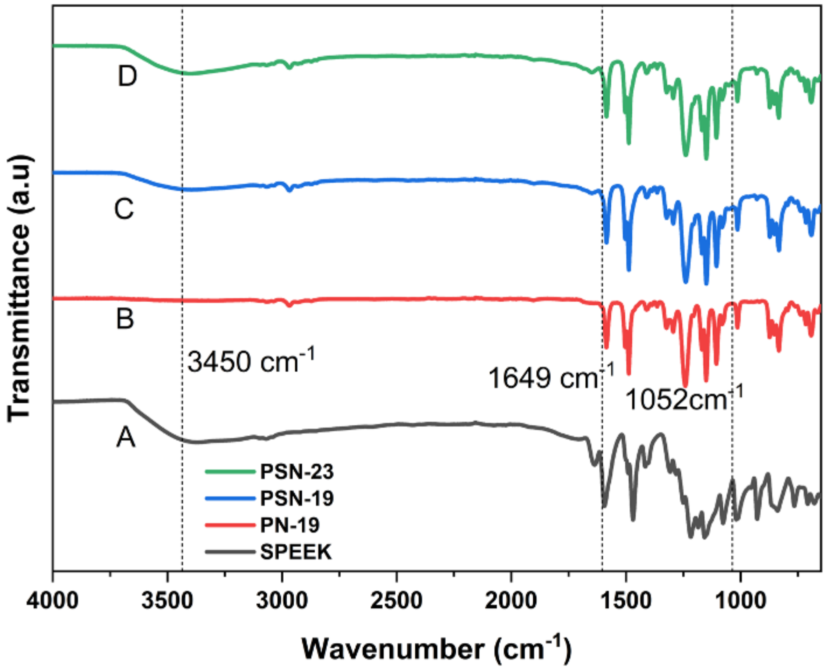

Figure S4. FTIR spectra showing the functional peaks comparison of fabricated membranes PN-19, PSN-19, and PSN-23.

Table S2. Elemental composition of C1s, O2s, S2p of PN-19, PSN-19, PSN-19O and PSN-19 (etched at 300s) using XPS.

	PN-19		PSN-19		PSN-19O	
Name	Peak BE	Atomic %	Peak BE	Atomic %	Peak BE	Atomic %
C1s	284.76	83.61	285.02	81.55	284.83	80.77
O2s	531.97	14.02	532.28	15.62	532.15	16.18
S2p	167.96	2.37	168.21	2.82	167.97	3.05

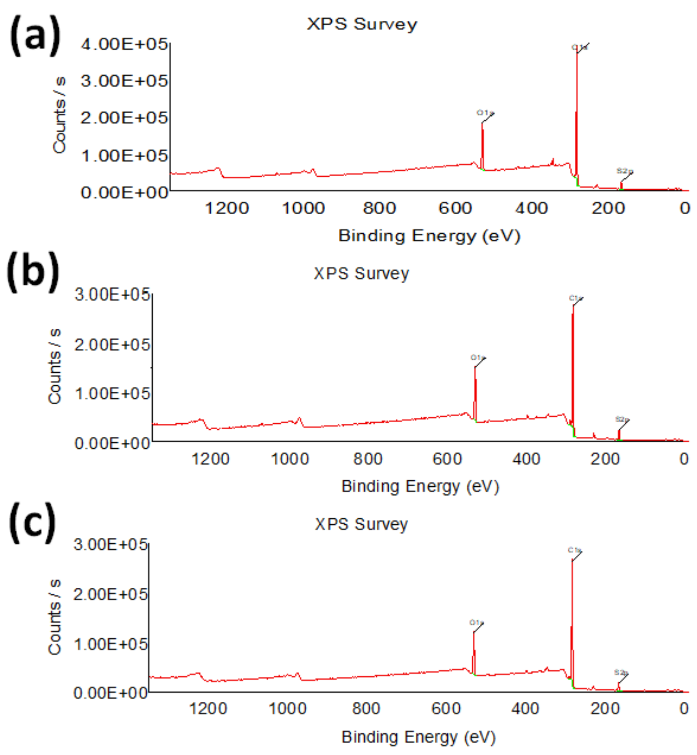

Figure S5. XPS characterization of membrane chemistry. XPS spectra of a) PN-19 b) PSN-19, and c) PSN-19O.

Morphologies of both surfaces and cross-sections of PSN-17 (lowest viscosity) and PSN-23 (highest) are shown in fig S6. PSN-17 membranes depict a porous surface. The viscosity of solution was lower and SPEEK addition enhanced the membrane porosity by rapid solvent-nonsolvent diffusion. Consequently, larger pores are formed resulting in a more permeable structure. PSN-23, shown in fig S6 (b), the surface became denser with fewer visible pores, and no pores were visible at lower magnifications. This dense structure is due to the increased viscosity of the casting solution at higher dope solution concentrations which caused greater polymeric chain entanglement and slower demixing of solvent-nonsolvent.

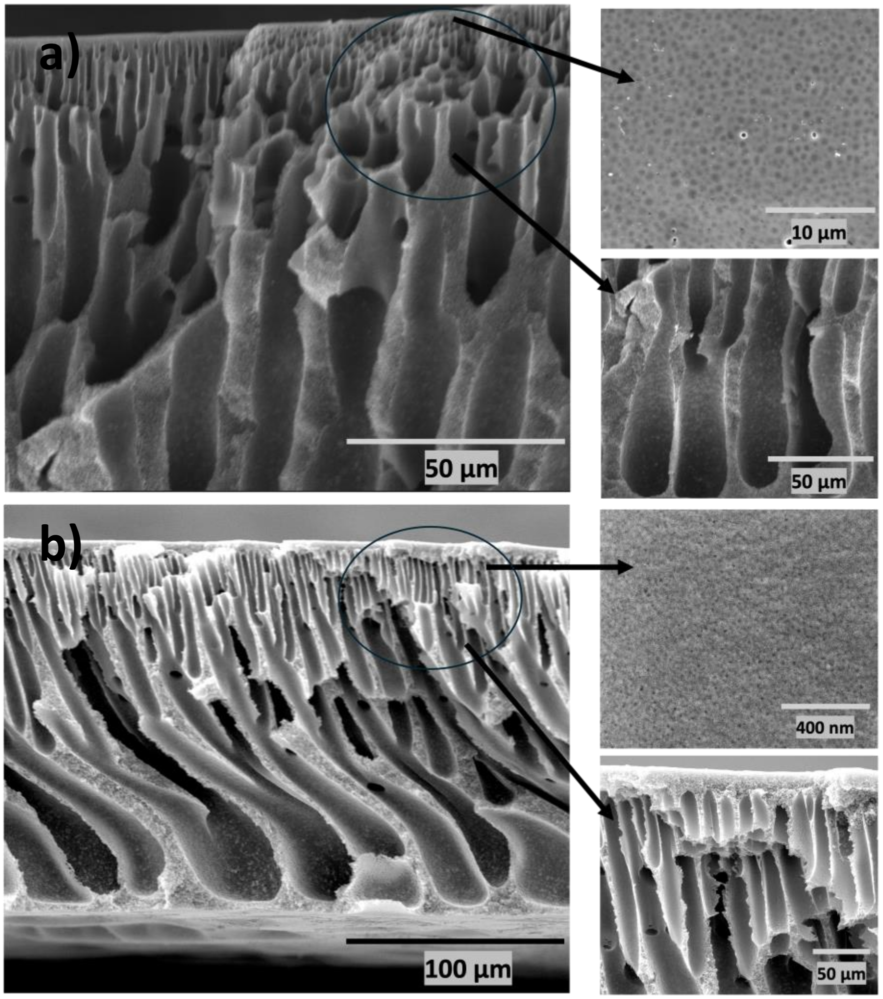

Figure S6. SEM cross-sectional and surface images of a) PSN-17 and b) PSN-23 membranes.

S.5.3 Total Nitrogen (TN) concentrations after filtration

Table S3. TN (mg/L) prior to and following filtration of MHRW at pH 7 through PSN-19 and PSN19W.

PSN-19	Unfiltered pH 7	Filtered 5x pH 7
Unannealed PSN-19	0.1538 ± 0.024	0.28155 ± 0.0795
Annealed PSN-19W	0.0906 ± 0.0316	0.1190 ± 0.0194

S.5.4 Membrane performance analysis

The filtration performance of PSf-SPEEK membranes was evaluated using various dyes (MB, CR, CV, AO2) in the water using a dead-end filtration cell. This finding would suggest that there are multiple factors that influence the removal of solute from water. While pore size is generally expected to control the rejection of organic solutes and the polymer framework density regulates water permeability, the incorporation of functional groups those contributing to charge density alters the separation dynamics. This modification enhances electrostatic interactions, disrupts solute diffusion pathways, and can further affect the membrane's selectivity and overall performance.

First, the MB was chosen as model dye to examine filtration properties, conducted in detail for polysulfone membranes named PN-17 and PN-19, and PSf-SPEEK membranes named PSN-17, PSN-19, PSN-21, PSN-23, as shown in fig S7 The PN-17 showed water flux of 67 LMH/bar, which decreased drastically as the concentration of the dope solution increased to 19% in PSN-19. Moreover, the PSf shows relatively hydrophobic as observed in contact angle measurement, which decreased the water permeability. The PN-17 showed an average rejection performance of 56%. As the concentration of the polysulfone membranes increased to 19% in PSN-19, the rejection did not improve much, although the permeability reduced to greater fraction. We incorporated the SPEEK in PSf to functionalize the membranes with sulfonic acid groups. The incorporation of SPEEK in PSN-17, PSN-19, PSN-21, and PSN-23 lead to increase in hydrophilicity and induced the negative charge on the surface of membranes due to presence of sulfonic acid groups. After being dope solution became more viscous with an increase in polymer concentration, the flux reduced due to denser structure. PSN-19 and PSN-21 showed less permeability than PSN-17, but the rejection was increased. With further increase in polymer concentration at PSN-23, the permeability reduced further but removal of MB increased too.

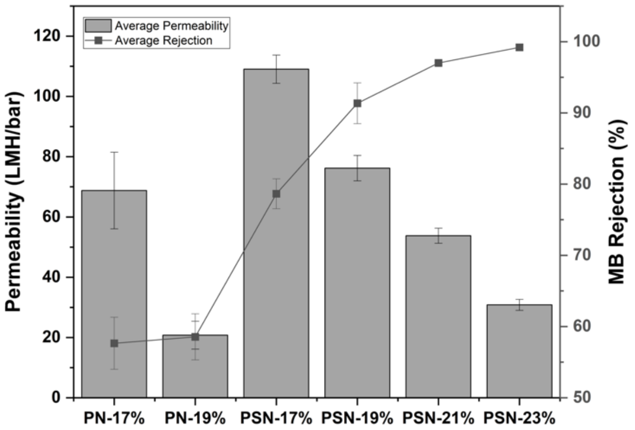

Figure S7. Average Permeability and rejection plot for MB dye with PN-17, PN-19, PSN-17, PSN-19, PSN-21, and PSN-23.

The average permeabilities and rejection for CR, CV, AO2 for PSN-19 and PSN-23 are shown in fig S8. Permeability and rejection Crystal Violet, a cationic dye, exhibited excellent rejection rates above 90% for both membranes, consistent with the membrane's negatively charged surface, which enhanced electrostatic interaction between the membrane and the dye molecules. This high rejection can also be linked to the role of SPEEK in the membrane composition. SPEEK introduced sulfonic acid groups, increasing the negative surface charge density and tightening the pore structure, which favors the rejection of larger, positively charged molecules like CV. CR is an anionic dye with relatively larger molecular weight showed average rejection above 75% in both PSN-19 and PSN-23. In contrast, AO2, a smaller anionic dye, showed the lowest rejection rates—18% for PSN-19 and 38% for PSN-23. The smaller molecular size of AO2 allowed it to permeate more easily through the membrane's pores, and since both the dye and membrane are negatively charged, electrostatic repulsion to assist in its rejection is not dominant. The presence of SPEEK played a less significant role in rejecting AO2 due to the weak interaction between the anionic dye and the negatively charged membrane surface. This shows that membrane performances are subject to type of solute and has a broad application prospect. The relative performance comparison is given in Table S4, and the relative comparison with other literature is presented in table S5.

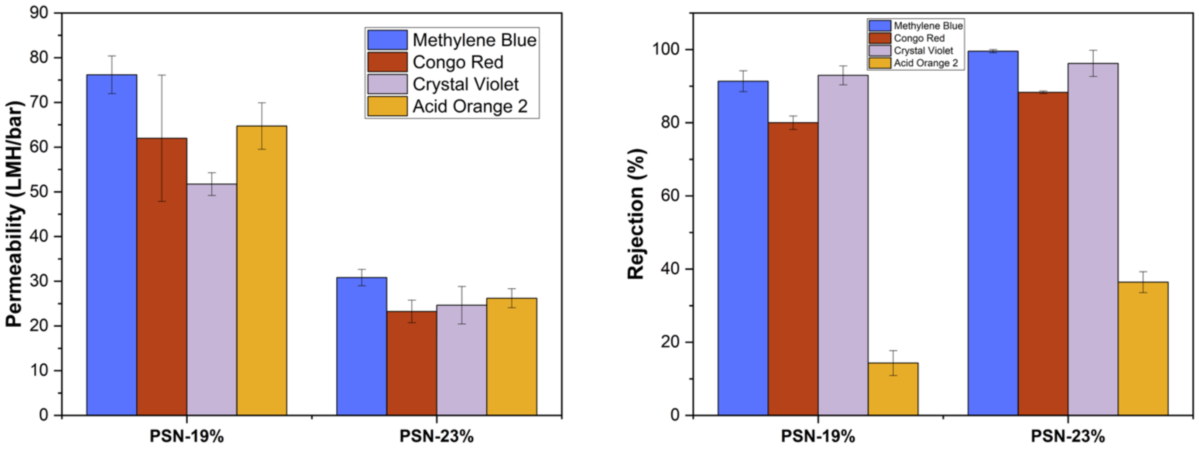

Figure S8. Average Permeability (left) and rejection data (right) of PSN-19 and PSN-23 membranes with MB, CR, CV, and AO2.

S.5.5. Mixed Dyes

The mixture of MB:AO2 was prepared at 50:50 ratio. Both are highly soluble in water and the UV-vis spectrum of the binary dye solution showed two distinct adsorption peaks at 483 and 663 nm. The intensity of the peaks was used to calculate %rejection of each of the dye. It is evident from the intensities that the MB gave higher rejections than AO2 due to different interaction with membrane surface.

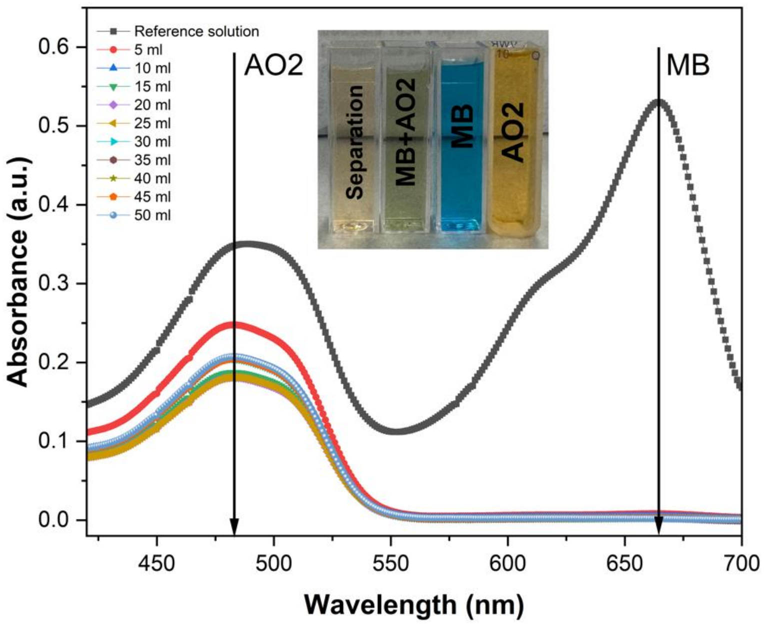

Figure S9. *UV-Vis spectra of Mixed dyes (MB + AO2) plotted after every 5mL of filtration.*

Supplementary References

1. Lyulin, A.V.; Sengupta, S.; Varughese, A.; Komarov, P.; Venkatnathan, A. Effect of annealing on structure and diffusion in hydrated Nafion membranes. *ACS Applied Polymer Materials* **2020**, *2*, 5058-5066.
2. Di Vona, M.L. Annealing of Polymer Membranes. In *Encyclopedia of Membranes*, Drioli, E., Giorno, L., Eds.; Springer Berlin Heidelberg: Berlin, Heidelberg, 2016; pp. 1-2.
3. Kusworo, T.D.; Kumoro, A.C.; Aryanti, N.; Utomo, D.P.; Qoyyimah; Hasbullah, H.; Alexandro, S.R. Effects of crosslinking and thermal annealing modifications on the performance of nanohybrid PSf-ZnO membranes for the treatment of raw and ozonated petroleum refinery wastewater. *Journal of Environmental Chemical Engineering* **2021**, *9*, 106200, doi:https://doi.org/10.1016/j.jece.2021.106200.
4. Kusworo, T.D.; Kumoro, A.C.; Aryanti, N.; Utomo, D.P.; Hasbullah, H.; Alexandro, S.R. Effects of crosslinking and thermal annealing modifications on the performance of nanohybrid PSf-ZnO membranes for the treatment of raw and ozonated petroleum refinery wastewater. *Journal of Environmental Chemical Engineering* **2021**, *9*, 106200.
5. Di Vona, M.L.; Sgreccia, E.; Licoccia, S.; Alberti, G.; Tortet, L.; Knauth, P. Analysis of temperature-promoted and solvent-assisted cross-linking in sulfonated poly (ether ether ketone)(SPEEK) proton-conducting membranes. *The Journal of Physical Chemistry B* **2009**, *113*, 7505-7512.
6. Jung, B.; Yoon, J.K.; Kim, B.; Rhee, H.-W. Effect of crystallization and annealing on polyacrylonitrile membranes for ultrafiltration. *Journal of membrane science* **2005**, *246*, 67-76.
7. Mavukkandy, M.O.; Bilad, M.R.; Giwa, A.; Hasan, S.W.; Arafat, H.A. Leaching of PVP from PVDF/PVP blend membranes: impacts on membrane structure and fouling in membrane bioreactors. *Journal of Materials Science* **2016**, *51*, 4328+.
8. O'Reilly, L.P.; Luke, C.J.; Perlmutter, D.H.; Silverman, G.A.; Pak, S.C. *C. elegans* in high-throughput drug discovery. *Adv Drug Deliv Rev* **2014**, *69-70*, 247-253, doi:10.1016/j.addr.2013.12.001.
9. Hunt, P.R. The *C. elegans* model in toxicity testing. *J Appl Toxicol* **2017**, *37*, 50-59, doi:10.1002/jat.3357.
10. Leuthner, T.C.; Zhang, S.; Kohrn, B.F.; Stapleton, H.M.; Baugh, L.R. Structure-specific variation in per- and polyfluoroalkyl substances toxicity among genetically diverse *Caenorhabditis elegans* strains. *bioRxiv* **2024**, doi:10.1101/2024.05.29.596269.
11. Cochran, J.P.; Unrine, J.M.; Coyne, M.; Tsyusko, O.V. Multiple stressor effects on a model soil nematode, *Caenorhabditis elegans*: Combined effects of the pathogen Klebsiella pneumoniae and zinc oxide nanoparticles. *Sci Total Environ* **2023**, *865*, 161307, doi:10.1016/j.scitotenv.2022.161307.
12. Sivasubramanian, G.; Hariharasubramanian, K.; Deivanayagam, P.; Ramaswamy, J. High-performance SPEEK/SWCNT/fly ash polymer electrolyte nanocomposite membranes for fuel cell applications. *Polymer Journal* **2017**, *49*, 703-709.
13. Fionah, A.; McLarney, K.; Judd, A.; Escobar, I.C. Effects of the Applied Potential on the Performance of Polysulfone Membranes Functionalized with Sulfonated Polyether Ether Ketone Polymers. *Membranes* **2023**, *13*, 675.
14. Anderson, L.J.; Yuan, X.; Fahs, G.B.; Moore, R.B. Blocky Ionomers via Sulfonation of Poly (ether ether ketone) in the Semicrystalline Gel State. *Macromolecules* **2018**, *51*, 6226-6237.
15. Ali, M.M.; Azam, A.; Rizvi, S. Synthesis and characterization of sulfonated poly ether ether ketone (SPEEK)/CNTs composite proton exchange membrane for application in fuel cells. *Materials Today: Proceedings* **2018**, *5*, 17901-17905.
16. Mahimai, B.M.; Sivasubramanian, G.; Sekar, K.; Kannaiyan, D.; Deivanayagam, P. Sulfonated poly (ether ether ketone): efficient ion-exchange polymer electrolytes for fuel cell applications–a versatile review. *Materials Advances* **2022**, *3*, 6085-6095.
17. Liu, X.; He, S.; Liu, S.; Jia, H.; Chen, L.; Zhang, B.; Zhang, L.; Lin, J. The roles of solvent type and amount of residual solvent on determining the structure and performance of sulfonated poly (ether ether ketone) proton exchange membranes. *Journal of Membrane Science* **2017**, *523*, 163-172.
18. Lau, W.-J.; Ismail, A. Effect of SPEEK content on the morphological and electrical properties of PES/SPEEK blend nanofiltration membranes. *Desalination* **2009**, *249*, 996-1005.

19. Parnian, M.J.; Rowshanzamir, S.; Gashoul, F. Comprehensive investigation of physicochemical and electrochemical properties of sulfonated poly (ether ether ketone) membranes with different degrees of sulfonation for proton exchange membrane fuel cell applications. *Energy* **2017**, *125*, 614-628.
20. Boom, R.; Van den Boomgaard, T.; Smolders, C. Equilibrium thermodynamics of a quaternary membrane-forming system with two polymers. 1. Calculations. *Macromolecules* **1994**, *27*, 2034-2040.
21. Chun, K.-Y.; Jang, S.-H.; Kim, H.-S.; Kim, Y.-W.; Han, H.-S.; Joe, Y.-i. Effects of solvent on the pore formation in asymmetric 6FDA–4, 4′ ODA polyimide membrane: terms of thermodynamics, precipitation kinetics, and physical factors. *Journal of Membrane Science* **2000**, *169*, 197-214.
22. Sterken, M.G.; Snoek, L.B.; Kammenga, J.E.; Andersen, E.C. The laboratory domestication of *Caenorhabditis elegans*. *Trends Genet* **2015**, *31*, 224-231, doi:10.1016/j.tig.2015.02.009.
23. Arata, Y.; Oshima, T.; Ikeda, Y.; Kimura, H.; Sako, Y. OP50, a bacterial strain conventionally used as food for laboratory maintenance of *C. elegans*, is a biofilm formation defective mutant. *MicroPubl Biol* **2020**, *2020*, doi:10.17912/micropub.biology.000216.
24. Cochran, J.P.; Ngy, P.; Unrine, J.M.; Matocha, C.J.; Tsyusko, O.V. Effects of Multiple Stressors, Pristine or Sulfidized Silver Nanomaterials, and a Pathogen on a Model Soil Nematode *Caenorhabditis elegans*. *Nanomaterials* **2024**, *14*, 913.
25. Ke, T.; Santamaría, A.; Tinkov, A.A.; Bornhorst, J.; Aschner, M. Generating Bacterial Foods in Toxicology Studies with *Caenorhabditis elegans*. *Current Protocols in Toxicology* **2020**, *84*, e94, doi:https://doi.org/10.1002/cptx.94.
26. Tsyusko, O.V.; Unrine, J.M.; Spurgeon, D.; Blalock, E.; Starnes, D.; Tseng, M.; Joice, G.; Bertsch, P.M. Toxicogenomic Responses of the Model Organism *Caenorhabditis elegans* to Gold Nanoparticles. *Environmental Science & Technology* **2012**, *46*, 4115-4124, doi:10.1021/es2033108.
27. Starnes, D.L.; Unrine, J.M.; Starnes, C.P.; Collin, B.E.; Oostveen, E.K.; Ma, R.; Lowry, G.V.; Bertsch, P.M.; Tsyusko, O.V. Impact of sulfidation on the bioavailability and toxicity of silver nanoparticles to *Caenorhabditis elegans*. *Environmental Pollution* **2015**, *196*, 239-246, doi:https://doi.org/10.1016/j.envpol.2014.10.009.
28. Starnes, D.L.; Lichtenberg, S.S.; Unrine, J.M.; Starnes, C.P.; Oostveen, E.K.; Lowry, G.V.; Bertsch, P.M.; Tsyusko, O.V. Distinct transcriptomic responses of *Caenorhabditis elegans* to pristine and sulfidized silver nanoparticles. *Environmental Pollution* **2016**, *213*, 314-321, doi:https://doi.org/10.1016/j.envpol.2016.01.020.
29. Cong, Y.; Yang, H.; Zhang, P.; Xie, Y.; Cao, X.; Zhang, L. Transcriptome Analysis of the Nematode *Caenorhabditis elegans* in Acidic Stress Environments. *Front Physiol* **2020**, *11*, 1107, doi:10.3389/fphys.2020.01107.
30. Bentahar, S.; Dbik, A.; El Khomri, M.; El Messaoudi, N.; Lacherai, A. Adsorption of methylene blue, crystal violet and congo red from binary and ternary systems with natural clay: Kinetic, isotherm, and thermodynamic. *Journal of Environmental Chemical Engineering* **2017**, *5*, 5921-5932.
31. Carvalho, S.S.; Carvalho, N.M. Degradation of organic dyes by water soluble iron (III) mononuclear complexes from bis-(2-pyridylmethyl) amine NNN-derivative ligands. *Inorganic Chemistry Communications* **2019**, *108*, 107507.
32. Information, N.C.f.B. PubChem Compound Summary for CID 135504738, Orange (II). Available online: https://pubchem.ncbi.nlm.nih.gov/compound/Orange-_II (accessed on October 24).
33. Saini, B.; Sinha, M.K.; Dey, A. Functionalized polymeric smart membrane for remediation of emerging environmental contaminants from industrial sources: Synthesis, characterization and potential applications. *Process Safety and Environmental Protection* **2022**, *161*, 684-702.
34. Hołda, A.K.; Aernouts, B.; Saeys, W.; Vankelecom, I.F. Study of polymer concentration and evaporation time as phase inversion parameters for polysulfone-based SRNF membranes. *Journal of membrane science* **2013**, *442*, 196-205.
35. Mazinani, S.; Darvishmanesh, S.; Ehsanzadeh, A.; Van der Bruggen, B. Phase separation analysis of Extem/solvent/non-solvent systems and relation with membrane morphology. *Journal of Membrane Science* **2017**, *526*, 301-314.
36. Han, M.-J.; Nam, S.-T. Thermodynamic and rheological variation in polysulfone solution by PVP and its effect in the preparation of phase inversion membrane. *Journal of Membrane Science* **2002**, *202*, 55-61.

37. Dong, X.; Shannon, H.D.; Parker, C.; De Jesus, S.; Escobar, I.C. Comparison of two low-hazard organic solvents as individual and cosolvents for the fabrication of polysulfone membranes. *AIChE Journal* **2020**, *66*, e16790.
38. Dlamini, D.S.; Matindi, C.; Vilakati, G.D.; Tesha, J.M.; Motsa, M.M.; Thwala, J.M.; Mamba, B.B.; Hoek, E.; Li, J. Fine-tuning the architecture of loose nanofiltration membrane for improved water flux, dye rejection and dye/salt selective separation. *Journal of Membrane Science* **2021**, *621*, 118930.
39. Shahmirzadi, M.A.A.; Hosseini, S.S.; Ruan, G.; Tan, N. Tailoring PES nanofiltration membranes through systematic investigations of prominent design, fabrication and operational parameters. *Rsc Advances* **2015**, *5*, 49080-49097.
40. Feng, Y.; Ren, J.; Li, H.; Zhao, D.; Sheng, L.; Wu, Y.; Zhao, W.; Deng, M. Effect of thermal annealing on gas separation performance and aggregation structures of block polyimide membranes. *Polymer* **2021**, *219*, 123538.
41. Zhang, G.; Li, Y.; Gao, A.; Zhang, Q.; Cui, J.; Zhao, S.; Zhan, X.; Yan, Y. Bio-inspired underwater superoleophobic PVDF membranes for highly-efficient simultaneous removal of insoluble emulsified oils and soluble anionic dyes. *Chemical Engineering Journal* **2019**, *369*, 576-587, doi:https://doi.org/10.1016/j.cej.2019.03.089.
42. Li, J.; Gong, J.-L.; Zeng, G.-M.; Zhang, P.; Song, B.; Cao, W.-C.; Fang, S.-Y.; Huan, S.-Y.; Ye, J. The performance of UiO-66-NH2/graphene oxide (GO) composite membrane for removal of differently charged mixed dyes. *Chemosphere* **2019**, *237*, 124517, doi:https://doi.org/10.1016/j.chemosphere.2019.124517.
43. Tahazadeh, S.; Mohammadi, T.; Tofighy, M.A.; Khanlari, S.; Karimi, H.; Emrooz, H.B.M. Development of cellulose acetate/metal-organic framework derived porous carbon adsorptive membrane for dye removal applications. *Journal of Membrane Science* **2021**, *638*, 119692.
44. Yun, J.; Wang, Y.; Liu, Z.; Li, Y.; Yang, H.; Xu, Z.-l. High efficient dye removal with hydrolyzed ethanolamine-Polyacrylonitrile UF membrane: Rejection of anionic dye and selective adsorption of cationic dye. *Chemosphere* **2020**, *259*, 127390.
45. Zheng, X.; Ni, C.; Xiao, W.; Liang, Y.; Li, Y. Ionic liquid grafted polyethersulfone nanofibrous membrane as recyclable adsorbent with simultaneous dye, heavy metal removal and antibacterial property. *Chemical Engineering Journal* **2022**, *428*, 132111.
46. Qi, Y.; Zhu, L.; Shen, X.; Sotto, A.; Gao, C.; Shen, J. Polythyleneimine-modified original positive charged nanofiltration membrane: Removal of heavy metal ions and dyes. *Separation and Purification Technology* **2019**, *222*, 117-124, doi:https://doi.org/10.1016/j.seppur.2019.03.083.
47. Koriem, O.A.; Kamel, A.M.; Shaaban, W.; Elkady, M.F. Enhancement of dye separation performance of eco-friendly cellulose acetate-based membranes. *Sustainability* **2022**, *14*, 14665.
48. Li, J.-H.; Zheng, H.; Lin, H.-X.; Zhang, B.-X.; Wang, J.-B.; Li, T.-L.; Zhang, Q.-Q. Preparation of three dimensional hydroxyapatite nanoparticles/poly (vinylidene fluoride) blend membranes with excellent dye removal efficiency and investigation of adsorption mechanism. *Chinese Journal of Polymer Science* **2019**, *37*, 1234-1247.
